# Supplementary material for: Inflammatory bone marrow signaling in pediatric acute myeloid leukemia distinguishes patients with poor outcomes
Source: Nat Commun. 2022 Nov 23;13:7186. doi: 10.1038/s41467-022-34965-4 (PMC9684530; doi:10.1038/s41467-022-34965-4)
Supplement: Supplementary file 1 — Supplementary Information [file 41467_2022_34965_MOESM1_ESM.pptx]

## Slide 1
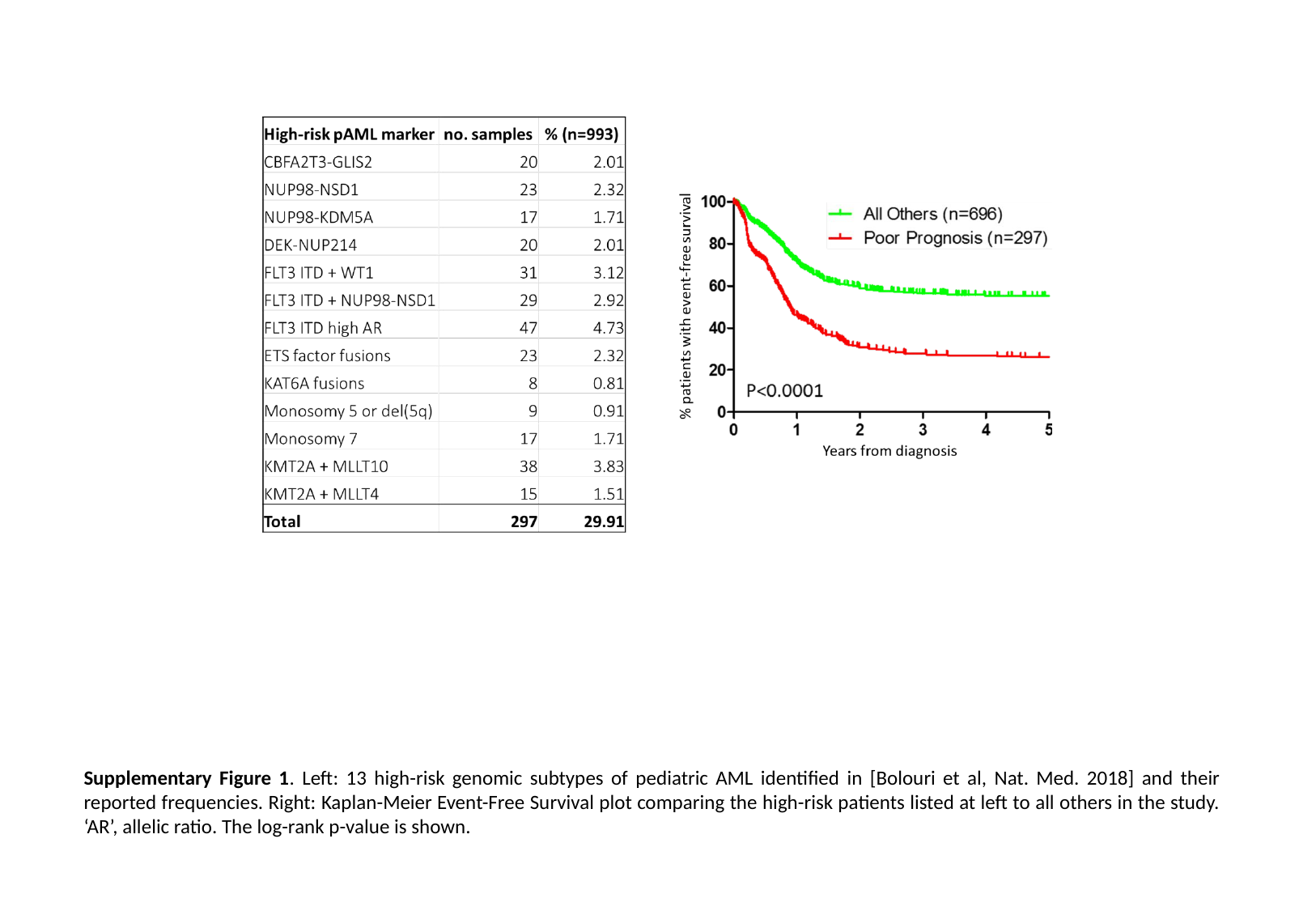

Supplementary Figure 1. Left: 13 high-risk genomic subtypes of pediatric AML identified in [Bolouri et al, Nat. Med. 2018] and their reported frequencies. Right: Kaplan-Meier Event-Free Survival plot comparing the high-risk patients listed at left to all others in the study. ‘AR’, allelic ratio. The log-rank p-value is shown.

## Slide 2
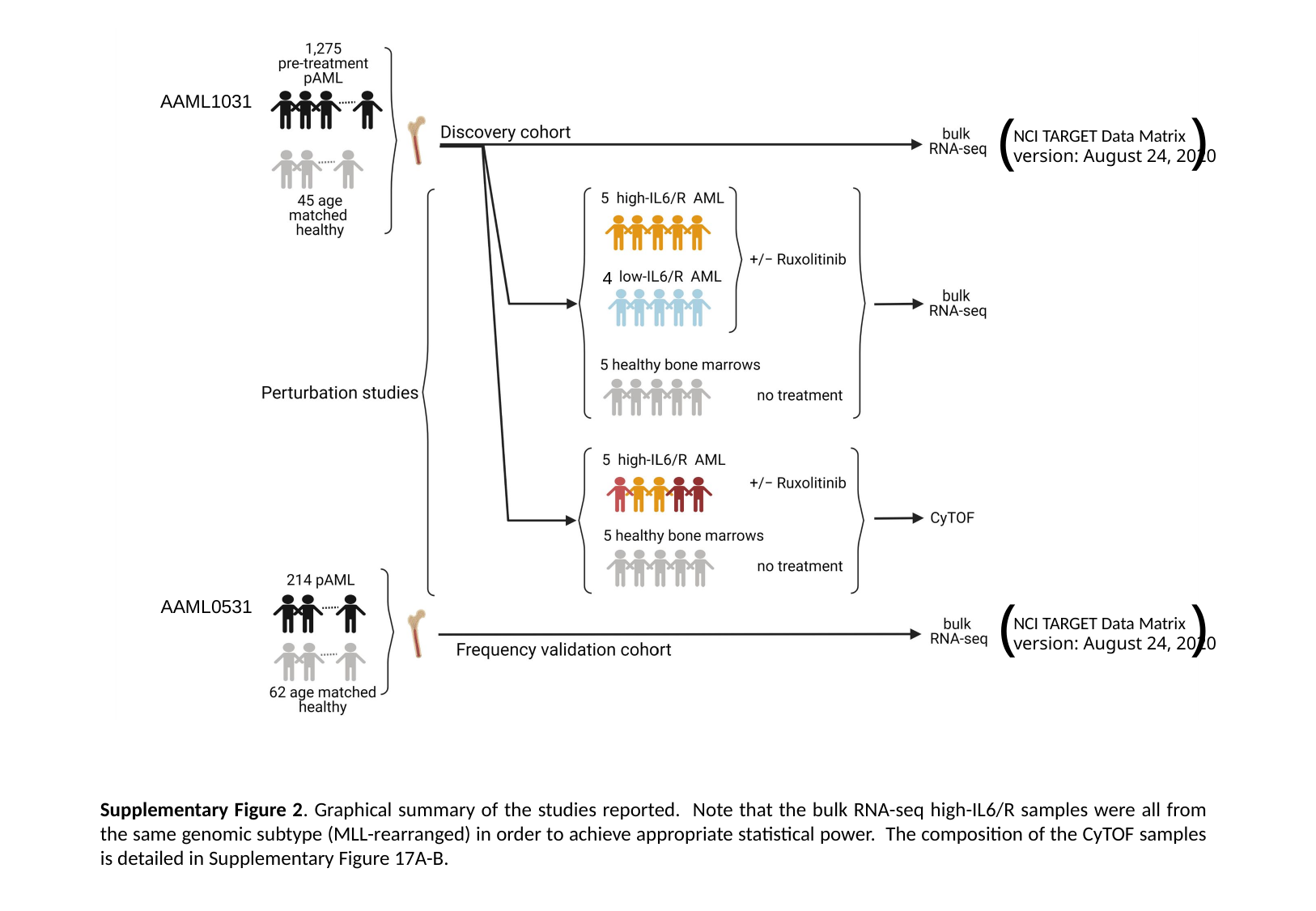

AAML1031
)
(
NCI TARGET Data Matrix
version: August 24, 2020
)
(
AAML0531
NCI TARGET Data Matrix
version: August 24, 2020
4
Supplementary Figure 2. Graphical summary of the studies reported. Note that the bulk RNA-seq high-IL6/R samples were all from the same genomic subtype (MLL-rearranged) in order to achieve appropriate statistical power. The composition of the CyTOF samples is detailed in Supplementary Figure 17A-B.

## Slide 3
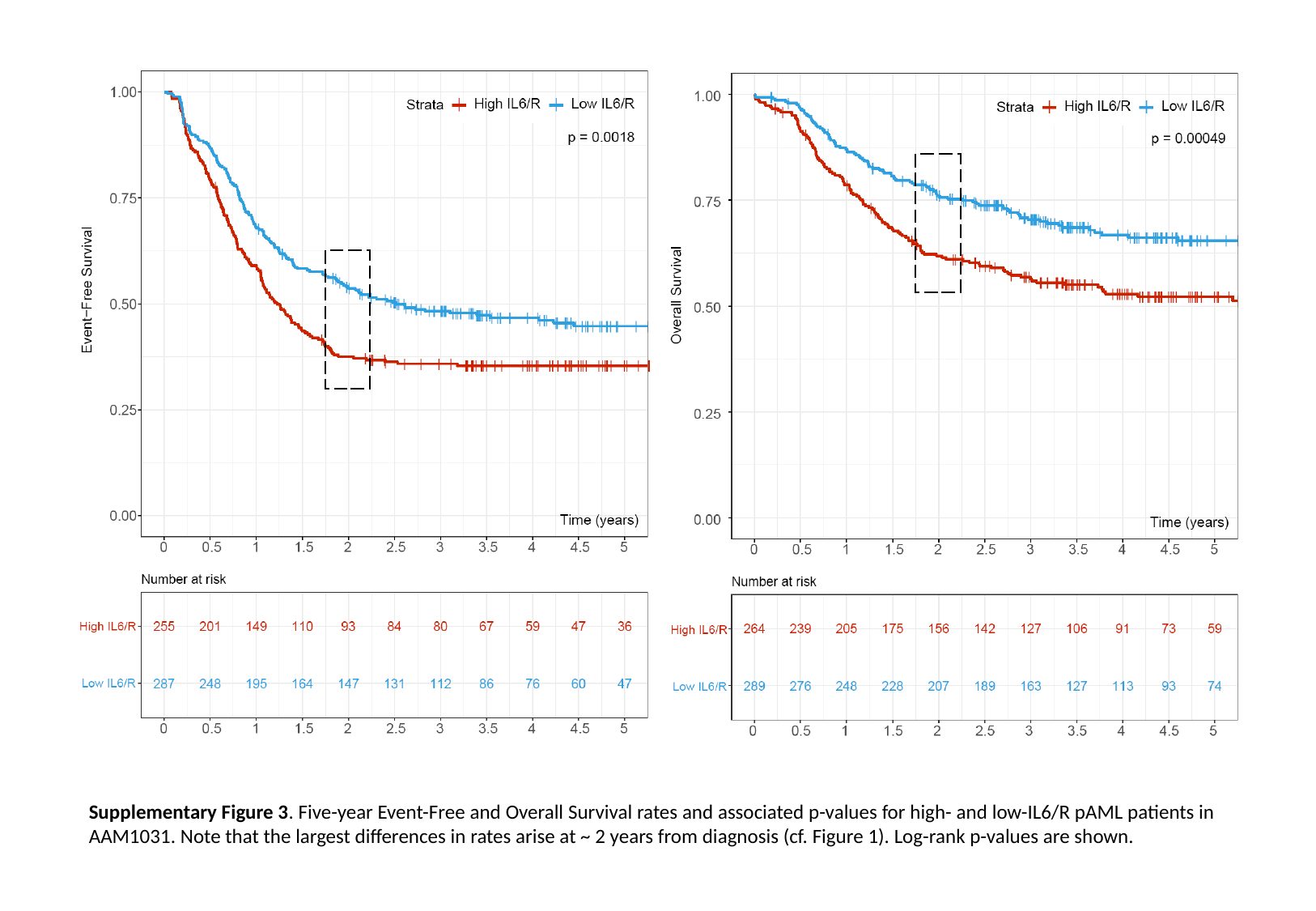

Supplementary Figure 3. Five-year Event-Free and Overall Survival rates and associated p-values for high- and low-IL6/R pAML patients in AAM1031. Note that the largest differences in rates arise at ~ 2 years from diagnosis (cf. Figure 1). Log-rank p-values are shown.

## Slide 4
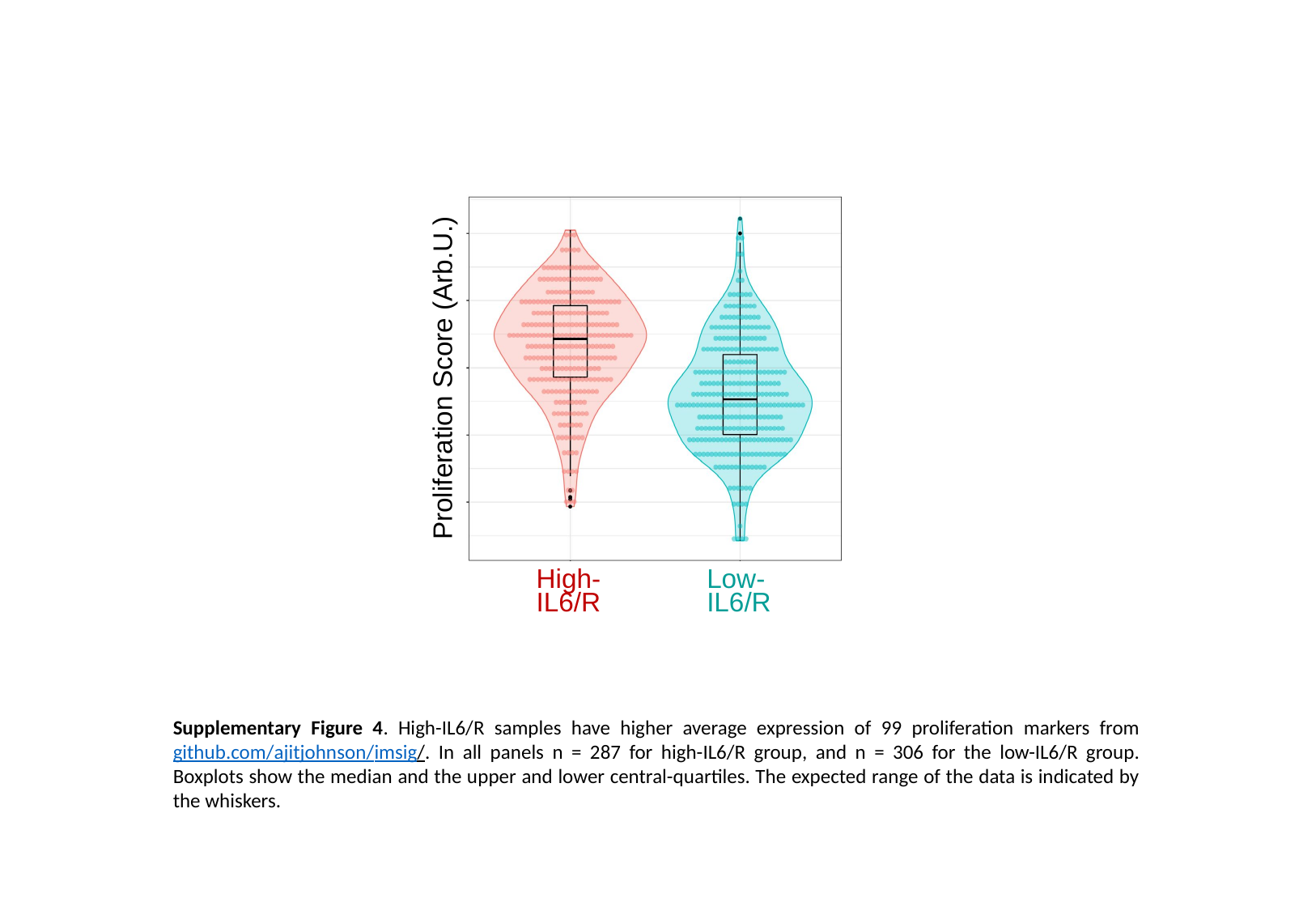

Proliferation Score (Arb.U.)
High-IL6/R
Low-IL6/R
Supplementary Figure 4. High-IL6/R samples have higher average expression of 99 proliferation markers from github.com/ajitjohnson/imsig/. In all panels n = 287 for high-IL6/R group, and n = 306 for the low-IL6/R group. Boxplots show the median and the upper and lower central-quartiles. The expected range of the data is indicated by the whiskers.

## Slide 5
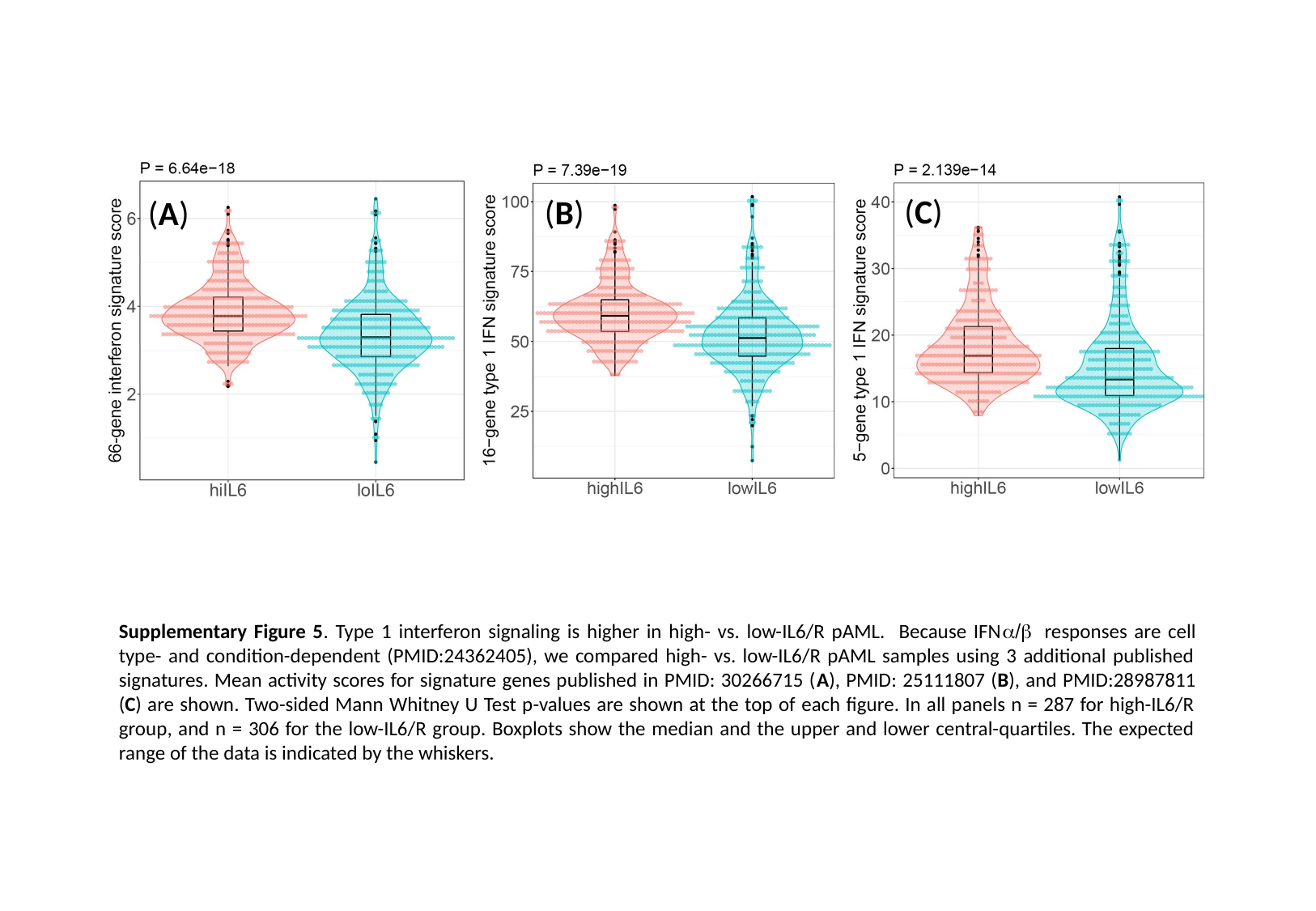

(C)
(B)
(A)
Supplementary Figure 5. Type 1 interferon signaling is higher in high- vs. low-IL6/R pAML. Because IFNa/b responses are cell type- and condition-dependent (PMID:24362405), we compared high- vs. low-IL6/R pAML samples using 3 additional published signatures. Mean activity scores for signature genes published in PMID: 30266715 (A), PMID: 25111807 (B), and PMID:28987811 (C) are shown. Two-sided Mann Whitney U Test p-values are shown at the top of each figure. In all panels n = 287 for high-IL6/R group, and n = 306 for the low-IL6/R group. Boxplots show the median and the upper and lower central-quartiles. The expected range of the data is indicated by the whiskers.

## Slide 6
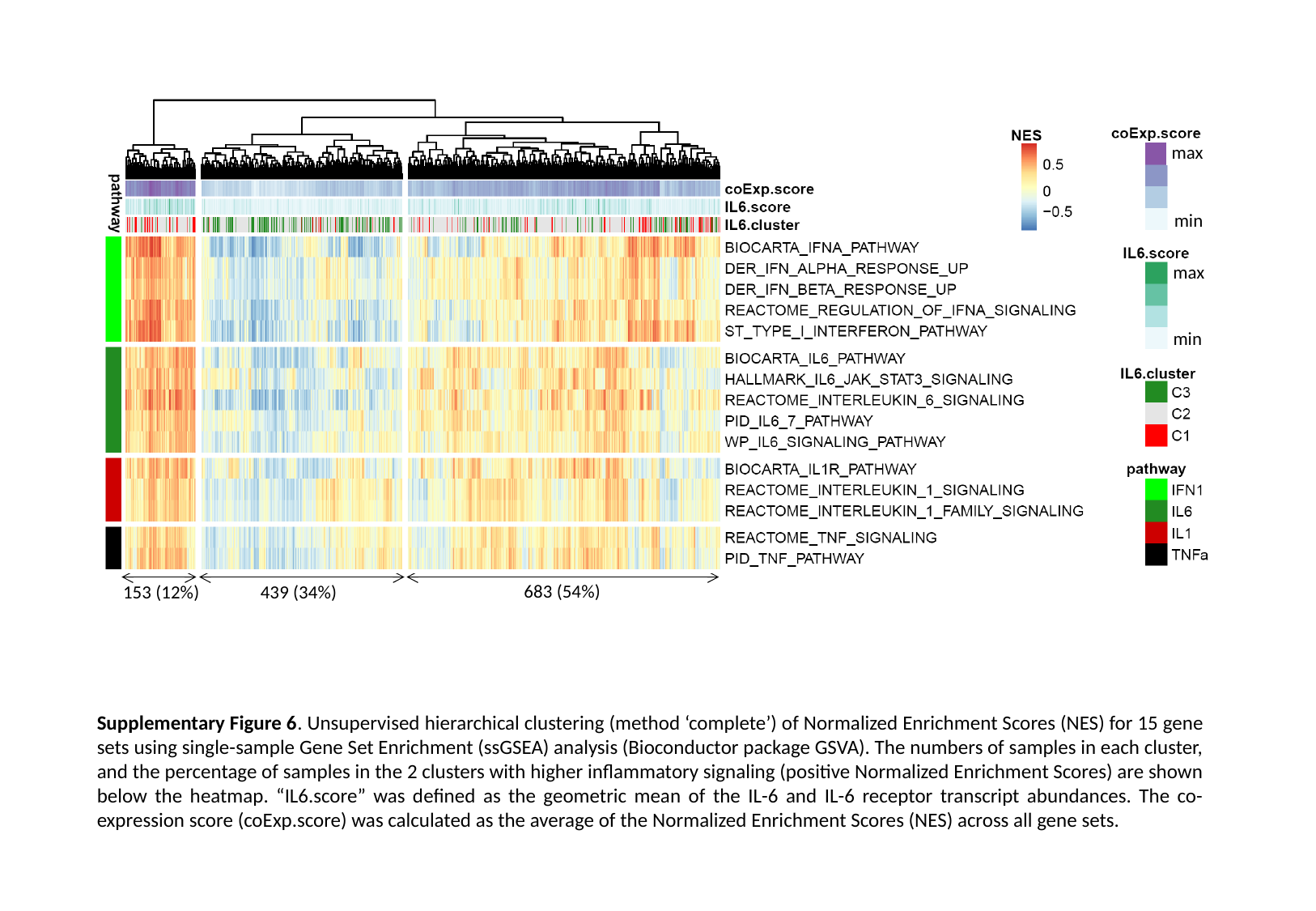

683 (54%)
439 (34%)
153 (12%)
max
min
max
min
Supplementary Figure 6. Unsupervised hierarchical clustering (method ‘complete’) of Normalized Enrichment Scores (NES) for 15 gene sets using single-sample Gene Set Enrichment (ssGSEA) analysis (Bioconductor package GSVA). The numbers of samples in each cluster, and the percentage of samples in the 2 clusters with higher inflammatory signaling (positive Normalized Enrichment Scores) are shown below the heatmap. “IL6.score” was defined as the geometric mean of the IL-6 and IL-6 receptor transcript abundances. The co-expression score (coExp.score) was calculated as the average of the Normalized Enrichment Scores (NES) across all gene sets.

## Slide 7
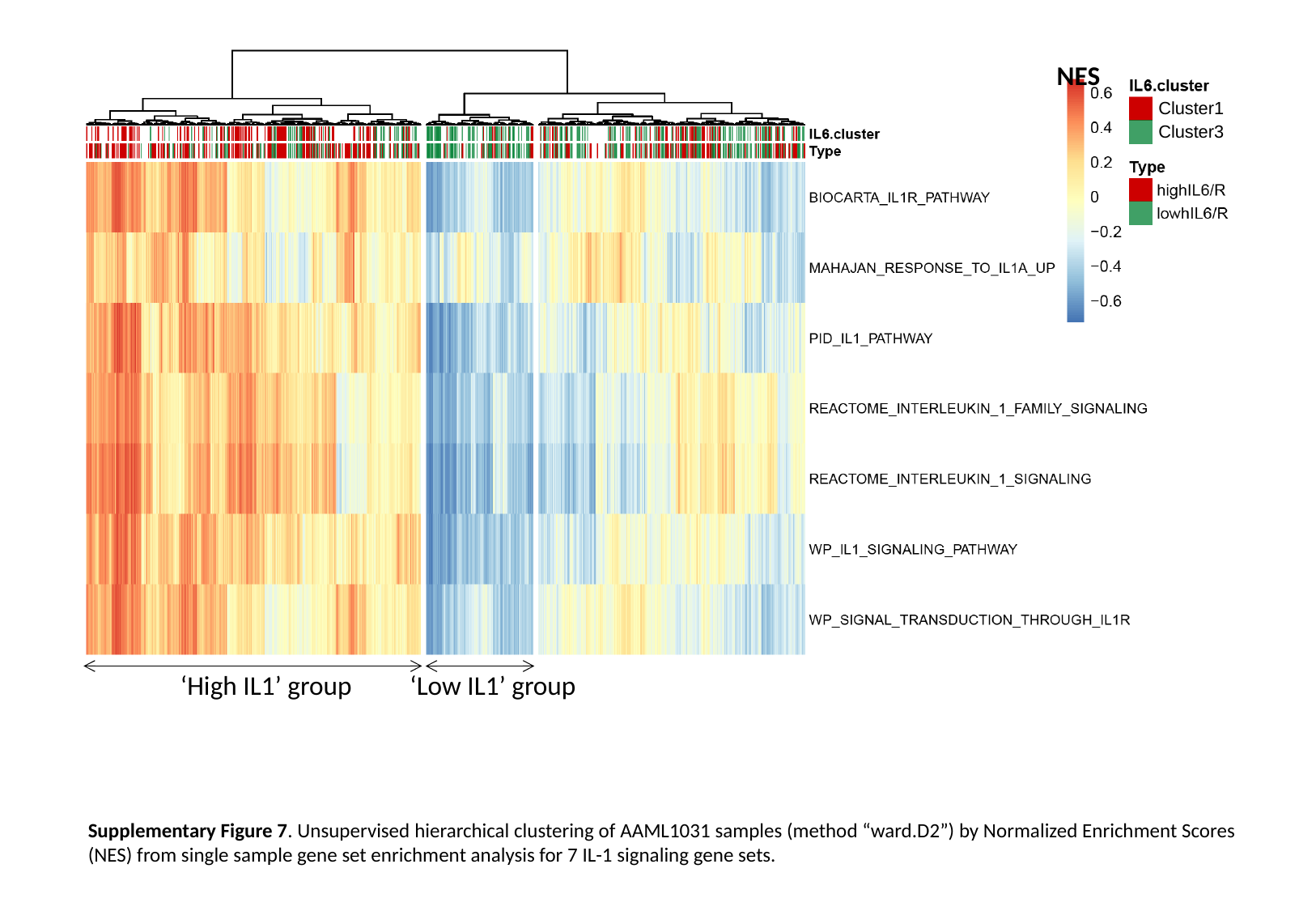

NES
‘High IL1’ group
‘Low IL1’ group
Cluster1
Cluster3
Supplementary Figure 7. Unsupervised hierarchical clustering of AAML1031 samples (method “ward.D2”) by Normalized Enrichment Scores (NES) from single sample gene set enrichment analysis for 7 IL-1 signaling gene sets.

## Slide 8
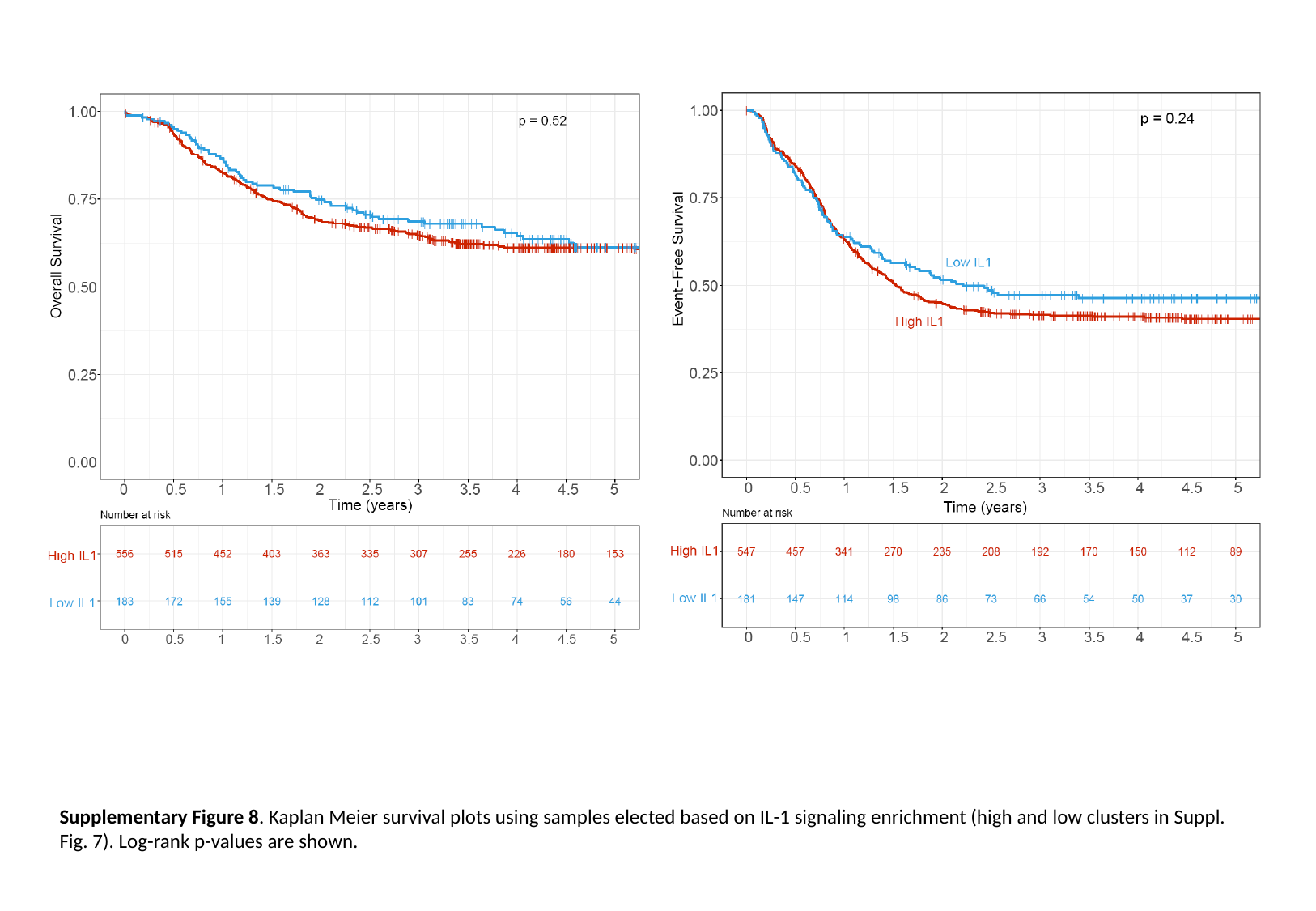

Supplementary Figure 8. Kaplan Meier survival plots using samples elected based on IL-1 signaling enrichment (high and low clusters in Suppl. Fig. 7). Log-rank p-values are shown.

## Slide 9
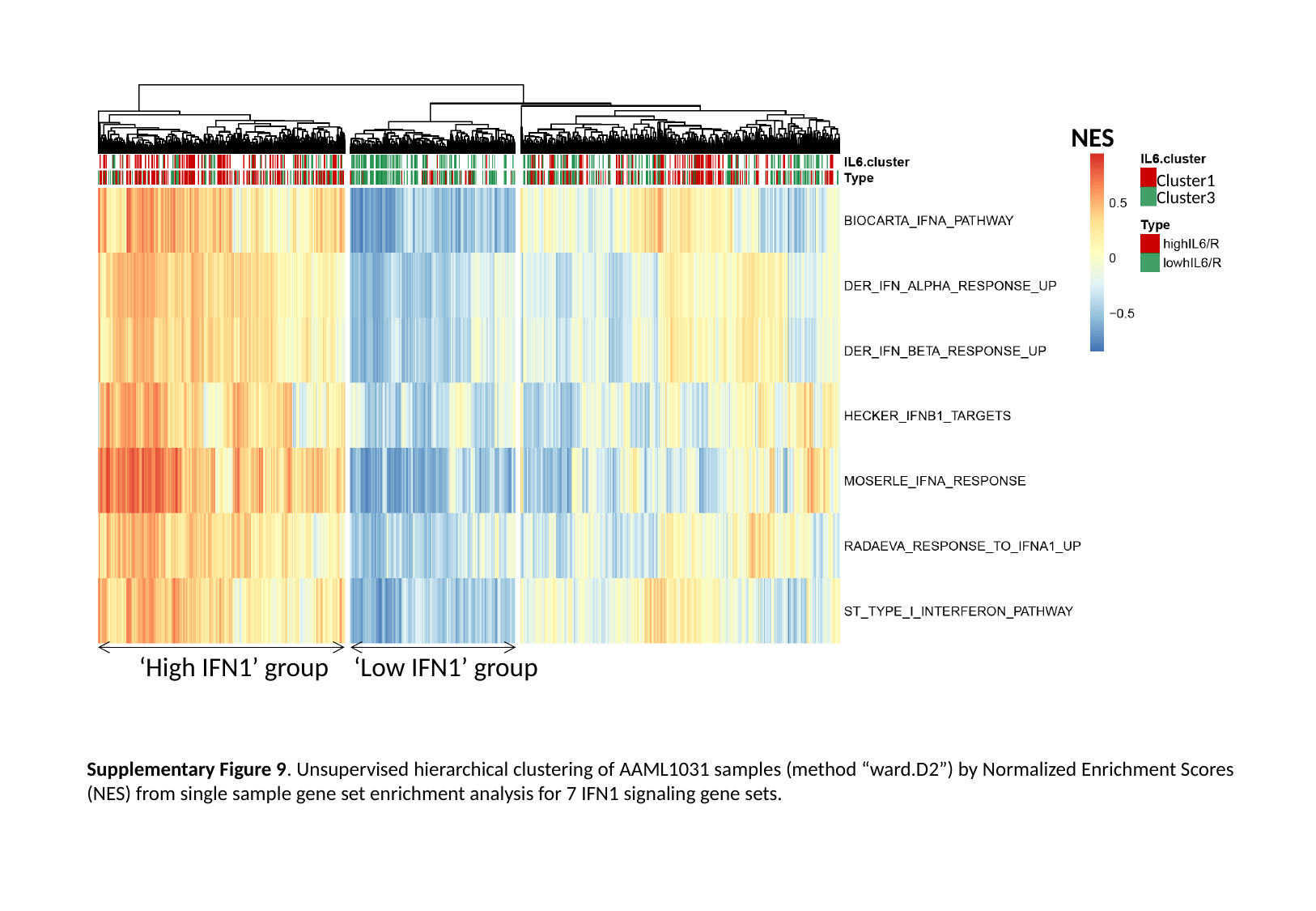

NES
‘High IFN1’ group
‘Low IFN1’ group
Cluster1
Cluster3
Supplementary Figure 9. Unsupervised hierarchical clustering of AAML1031 samples (method “ward.D2”) by Normalized Enrichment Scores (NES) from single sample gene set enrichment analysis for 7 IFN1 signaling gene sets.

## Slide 10
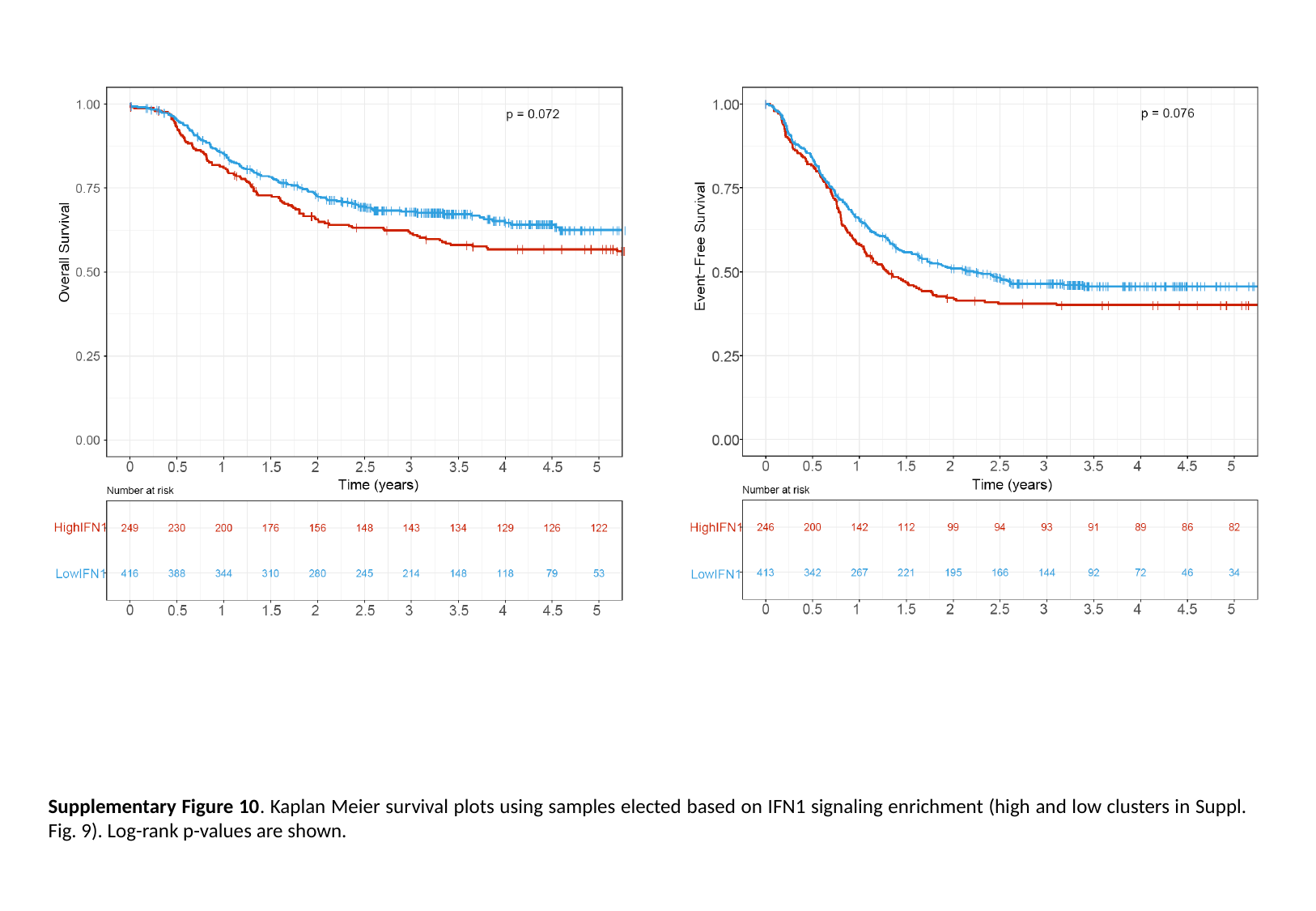

Supplementary Figure 10. Kaplan Meier survival plots using samples elected based on IFN1 signaling enrichment (high and low clusters in Suppl. Fig. 9). Log-rank p-values are shown.

## Slide 11
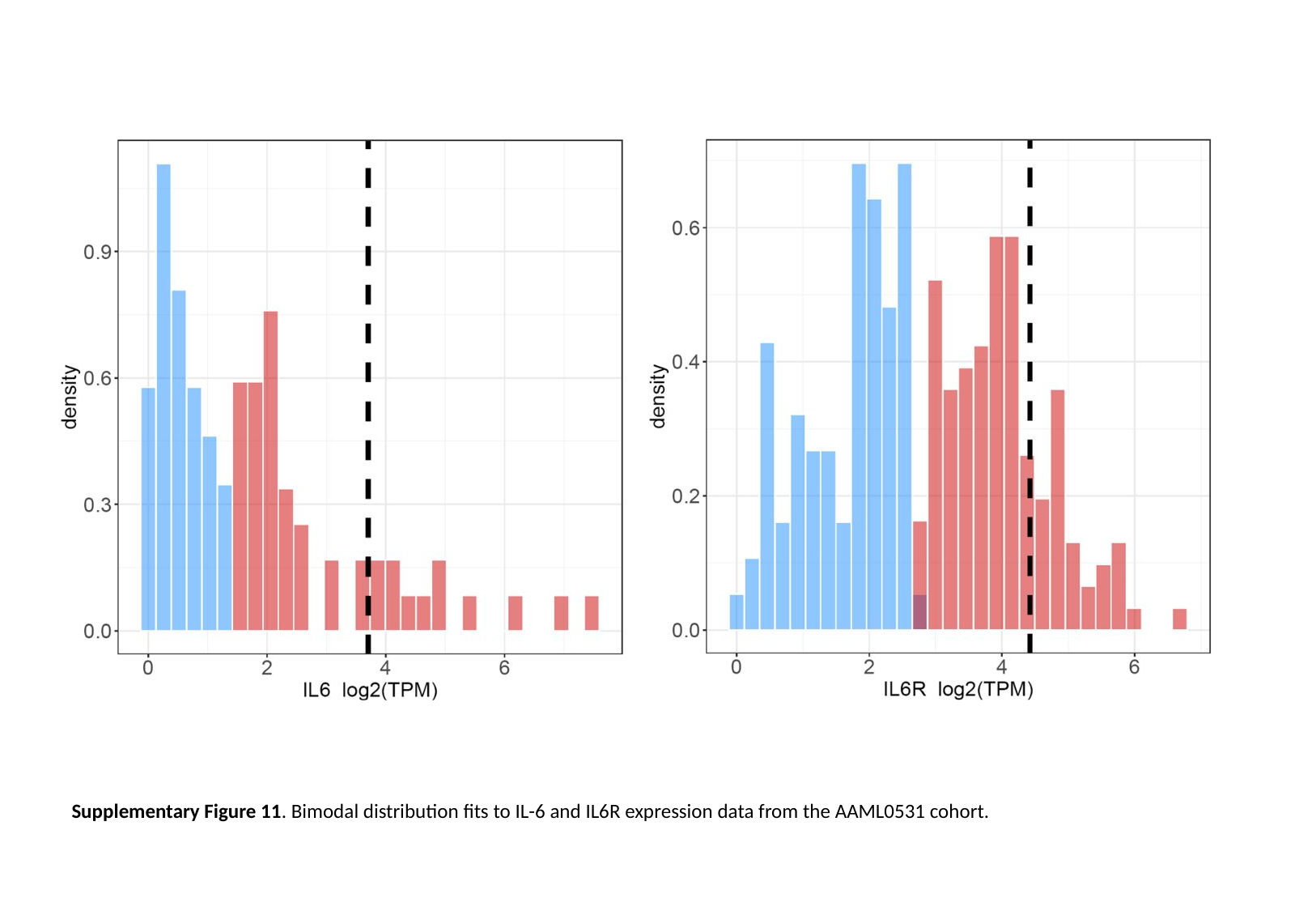

Supplementary Figure 11. Bimodal distribution fits to IL-6 and IL6R expression data from the AAML0531 cohort.

## Slide 12
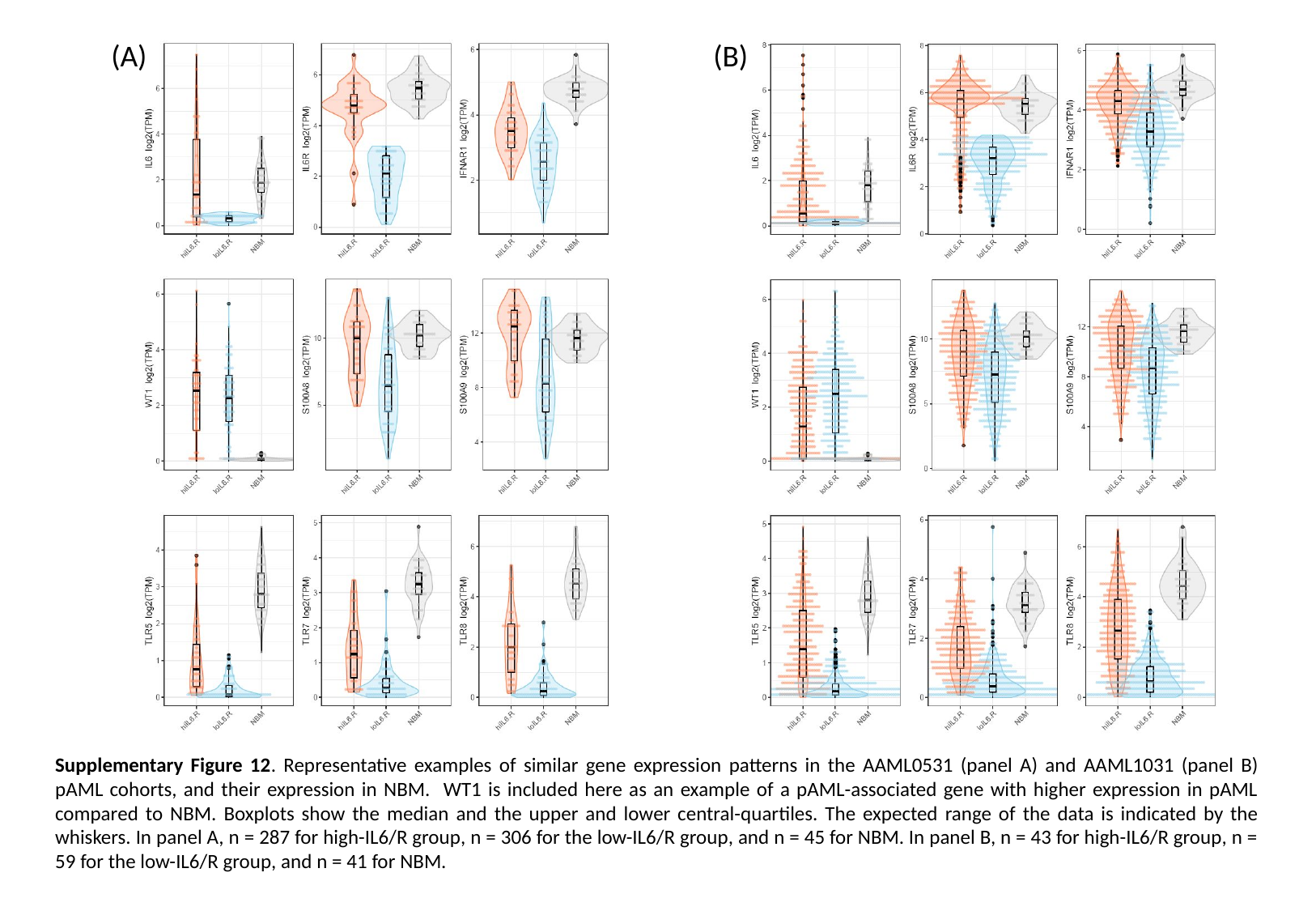

(A)
(B)
Supplementary Figure 12. Representative examples of similar gene expression patterns in the AAML0531 (panel A) and AAML1031 (panel B) pAML cohorts, and their expression in NBM. WT1 is included here as an example of a pAML-associated gene with higher expression in pAML compared to NBM. Boxplots show the median and the upper and lower central-quartiles. The expected range of the data is indicated by the whiskers. In panel A, n = 287 for high-IL6/R group, n = 306 for the low-IL6/R group, and n = 45 for NBM. In panel B, n = 43 for high-IL6/R group, n = 59 for the low-IL6/R group, and n = 41 for NBM.

## Slide 13
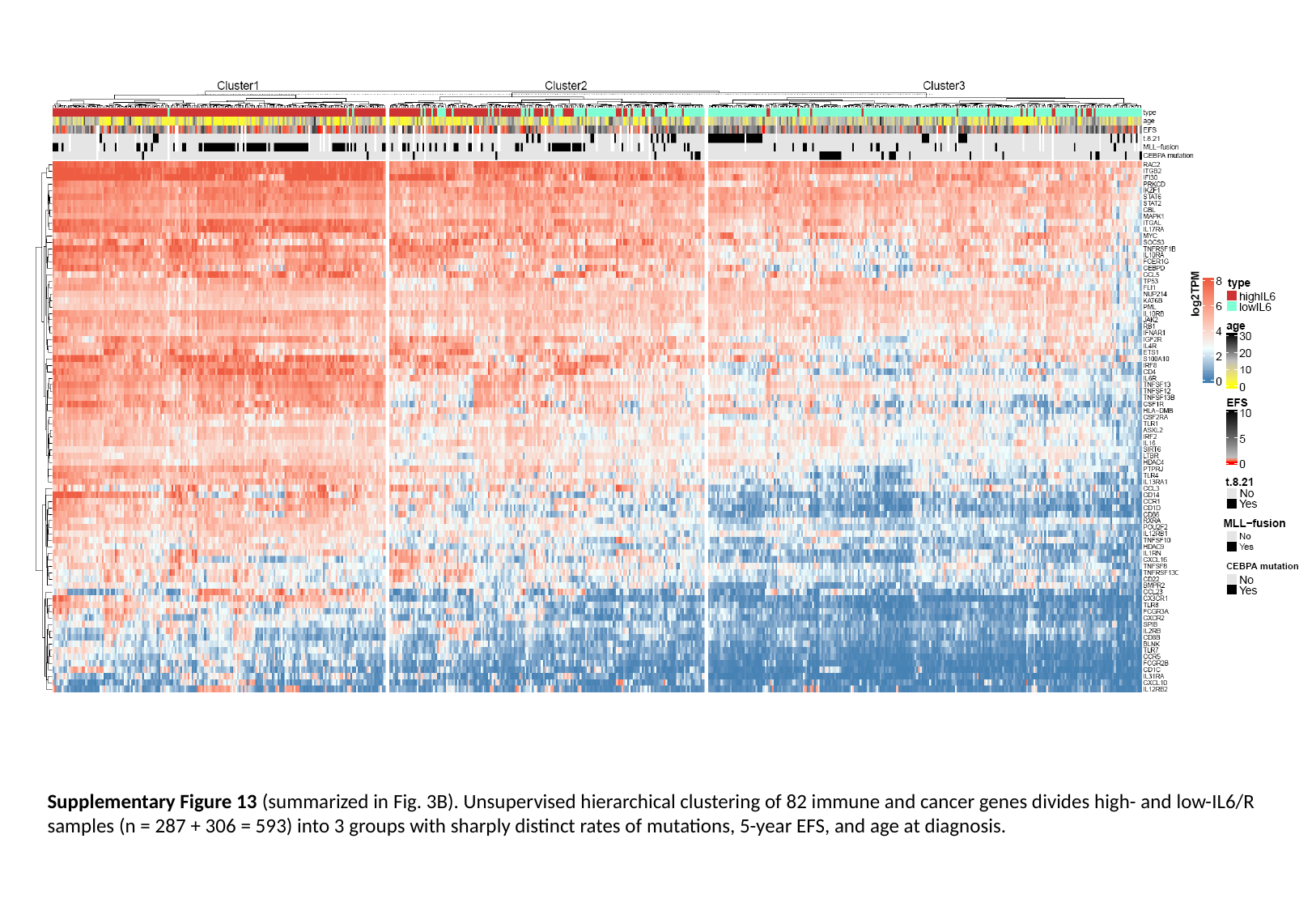

Supplementary Figure 13 (summarized in Fig. 3B). Unsupervised hierarchical clustering of 82 immune and cancer genes divides high- and low-IL6/R samples (n = 287 + 306 = 593) into 3 groups with sharply distinct rates of mutations, 5-year EFS, and age at diagnosis.

## Slide 14
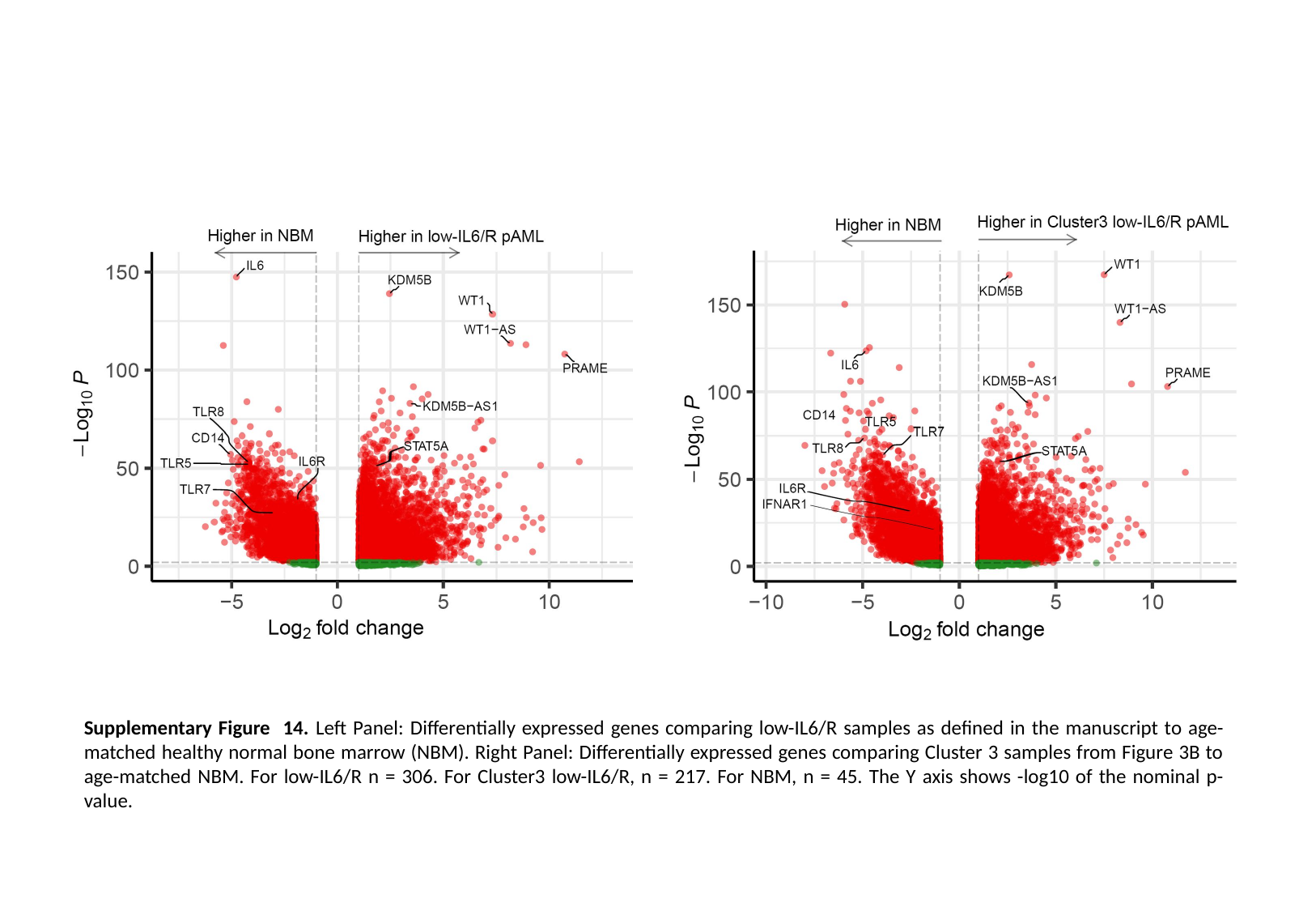

Supplementary Figure 14. Left Panel: Differentially expressed genes comparing low-IL6/R samples as defined in the manuscript to age-matched healthy normal bone marrow (NBM). Right Panel: Differentially expressed genes comparing Cluster 3 samples from Figure 3B to age-matched NBM. For low-IL6/R n = 306. For Cluster3 low-IL6/R, n = 217. For NBM, n = 45. The Y axis shows -log10 of the nominal p-value.

## Slide 15
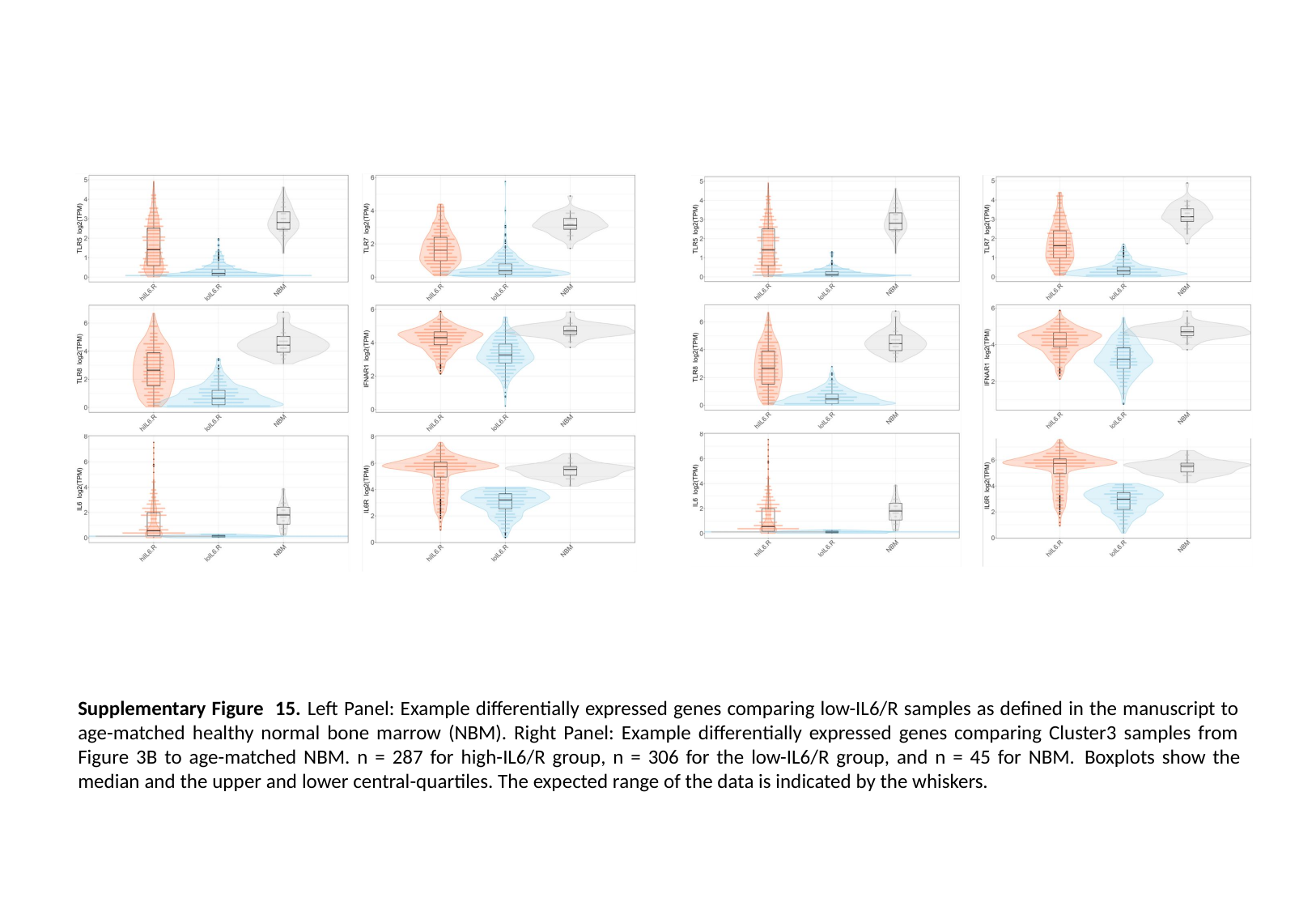

Supplementary Figure 15. Left Panel: Example differentially expressed genes comparing low-IL6/R samples as defined in the manuscript to age-matched healthy normal bone marrow (NBM). Right Panel: Example differentially expressed genes comparing Cluster3 samples from Figure 3B to age-matched NBM. n = 287 for high-IL6/R group, n = 306 for the low-IL6/R group, and n = 45 for NBM. Boxplots show the median and the upper and lower central-quartiles. The expected range of the data is indicated by the whiskers.

## Slide 16
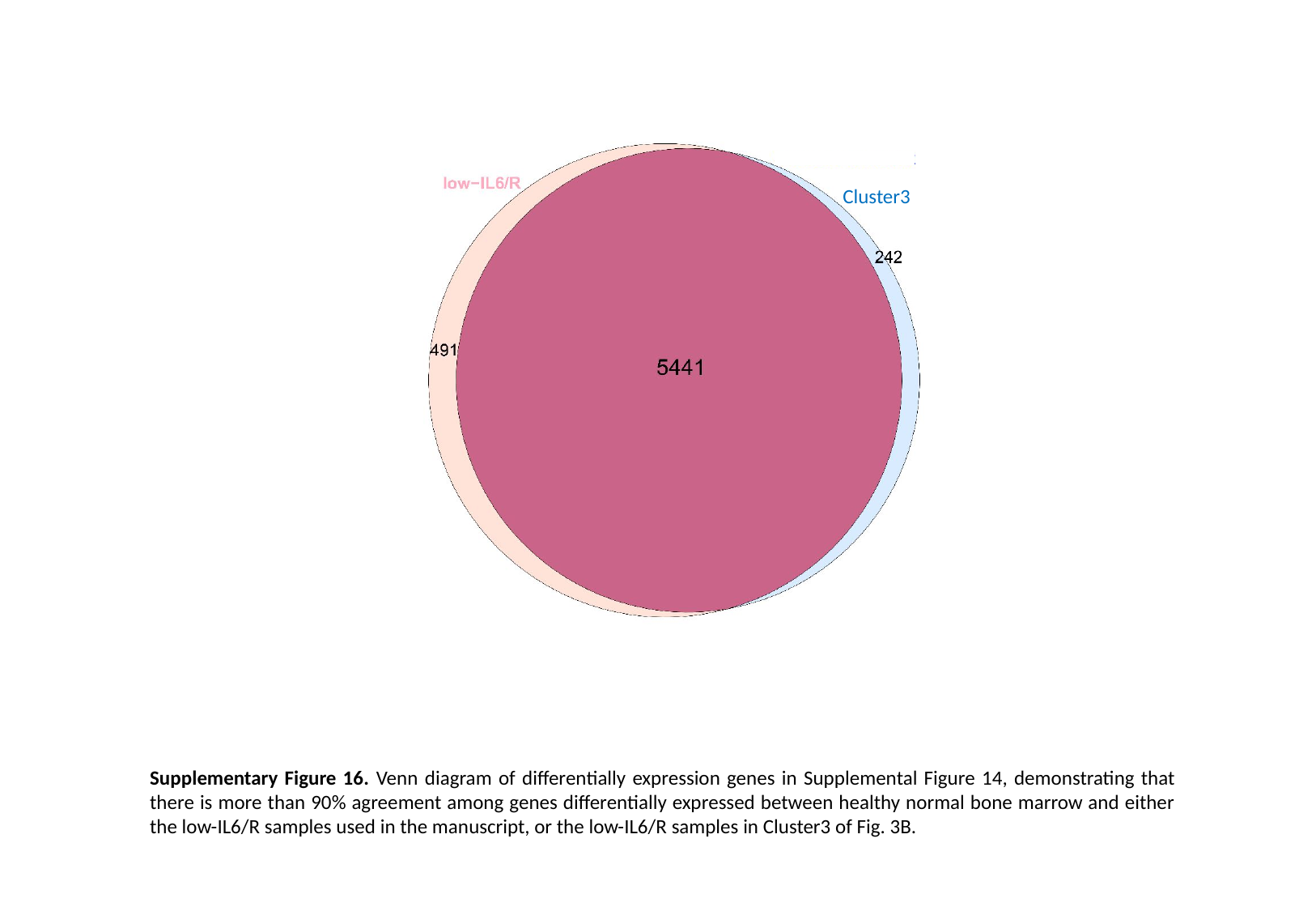

Cluster3
Supplementary Figure 16. Venn diagram of differentially expression genes in Supplemental Figure 14, demonstrating that there is more than 90% agreement among genes differentially expressed between healthy normal bone marrow and either the low-IL6/R samples used in the manuscript, or the low-IL6/R samples in Cluster3 of Fig. 3B.

## Slide 17
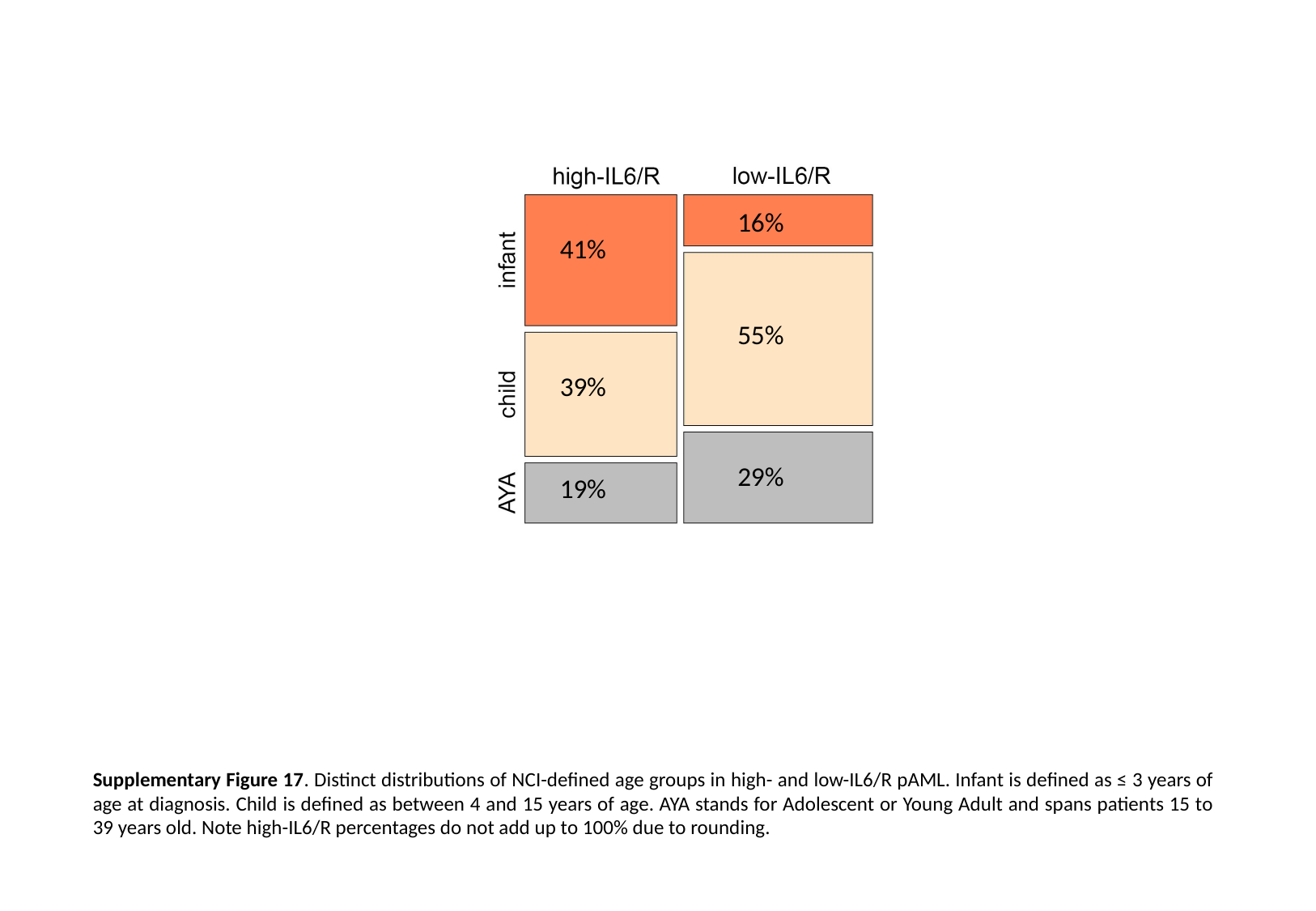

16%
41%
55%
39%
29%
19%
Supplementary Figure 17. Distinct distributions of NCI-defined age groups in high- and low-IL6/R pAML. Infant is defined as ≤ 3 years of age at diagnosis. Child is defined as between 4 and 15 years of age. AYA stands for Adolescent or Young Adult and spans patients 15 to 39 years old. Note high-IL6/R percentages do not add up to 100% due to rounding.

## Slide 18
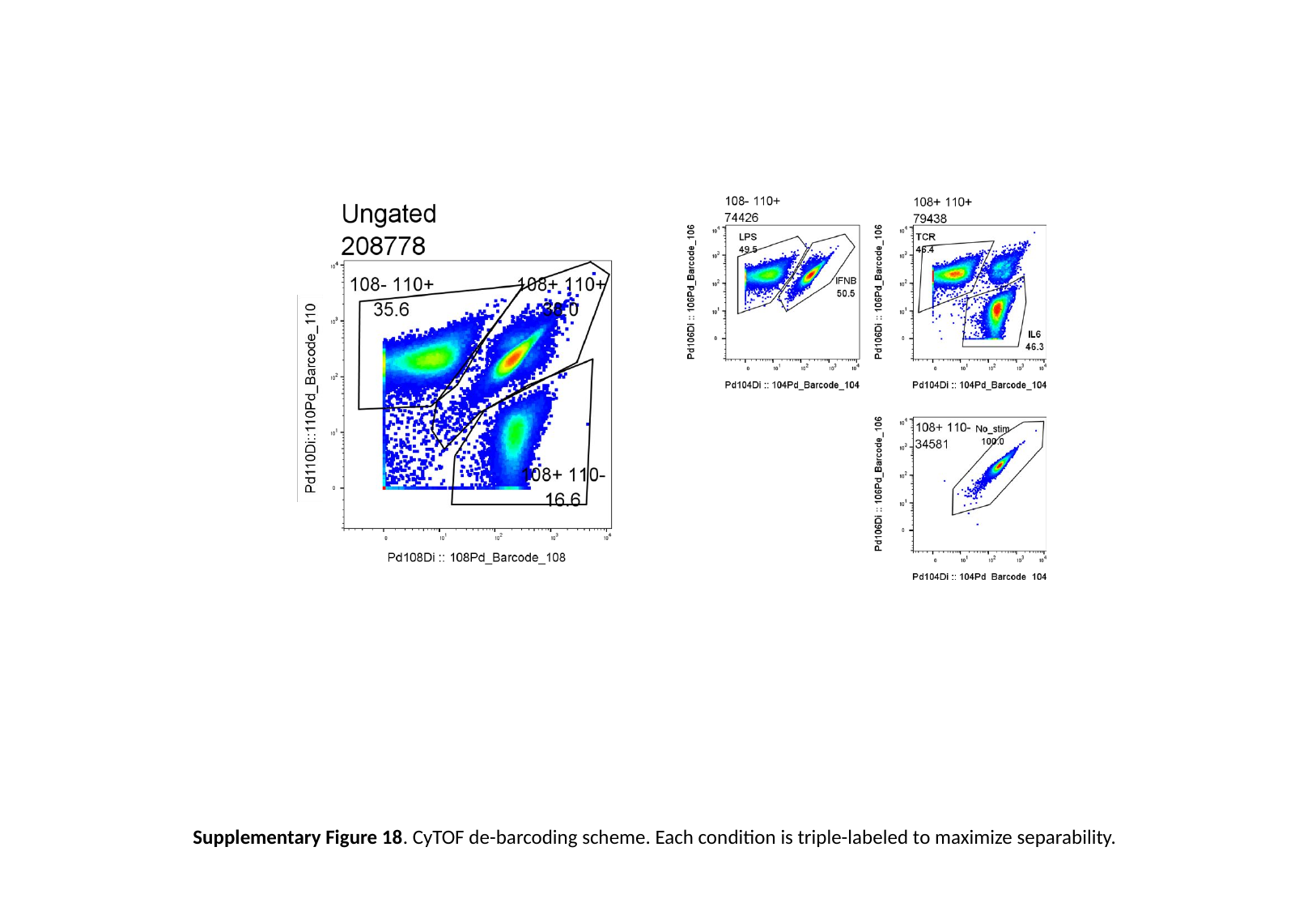

Supplementary Figure 18. CyTOF de-barcoding scheme. Each condition is triple-labeled to maximize separability.

## Slide 19
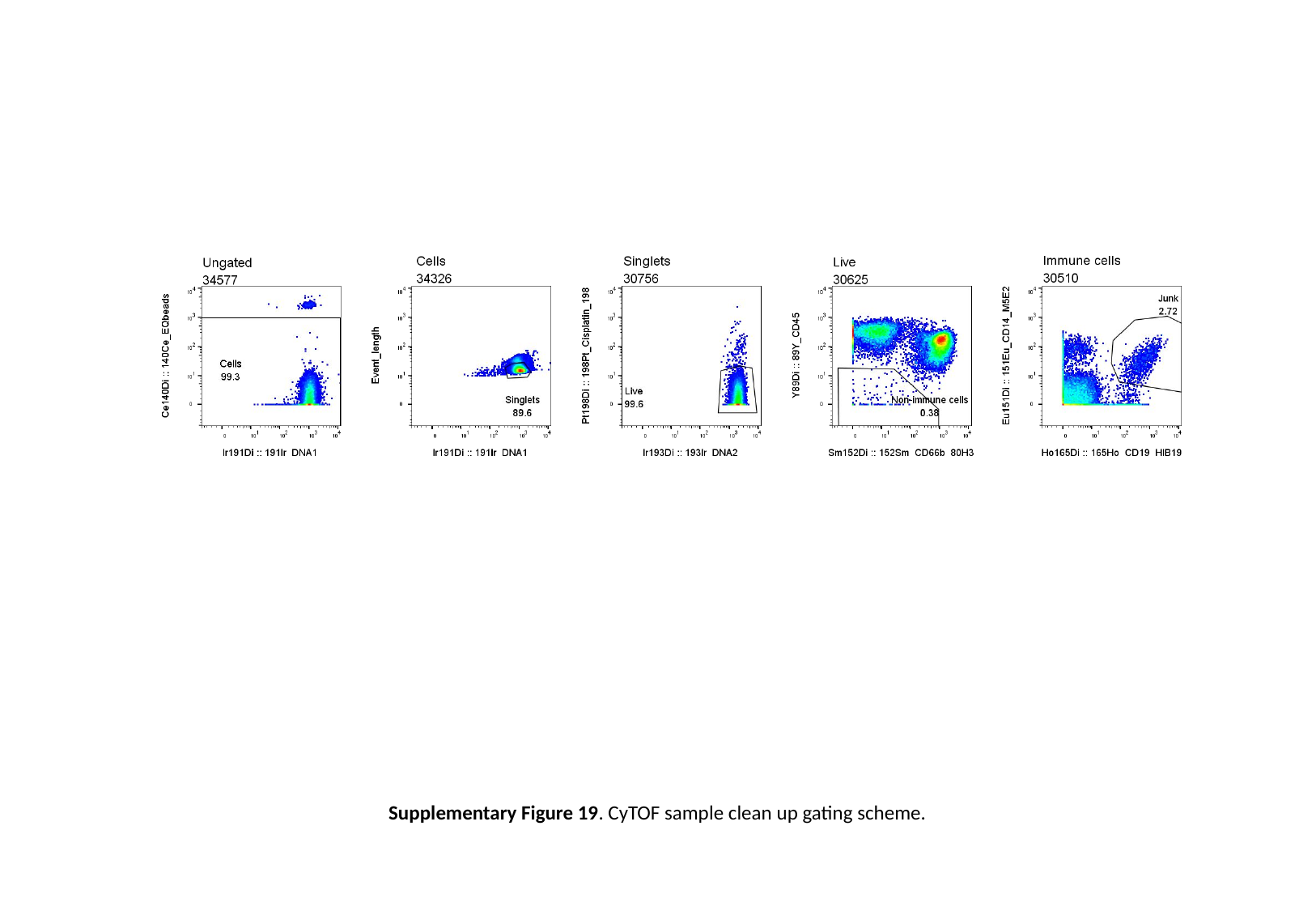

Supplementary Figure 19. CyTOF sample clean up gating scheme.

## Slide 20
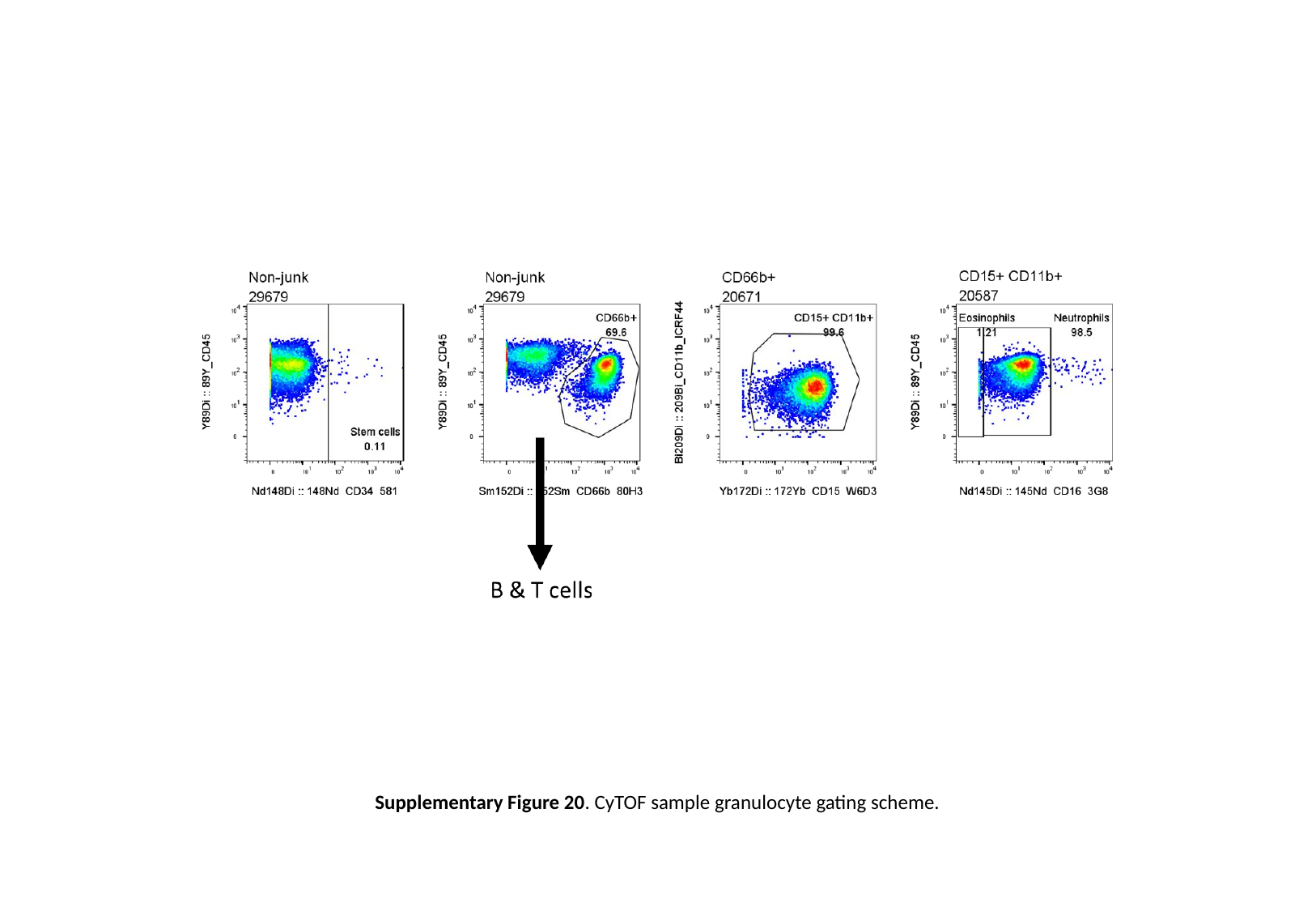

Supplementary Figure 20. CyTOF sample granulocyte gating scheme.

## Slide 21
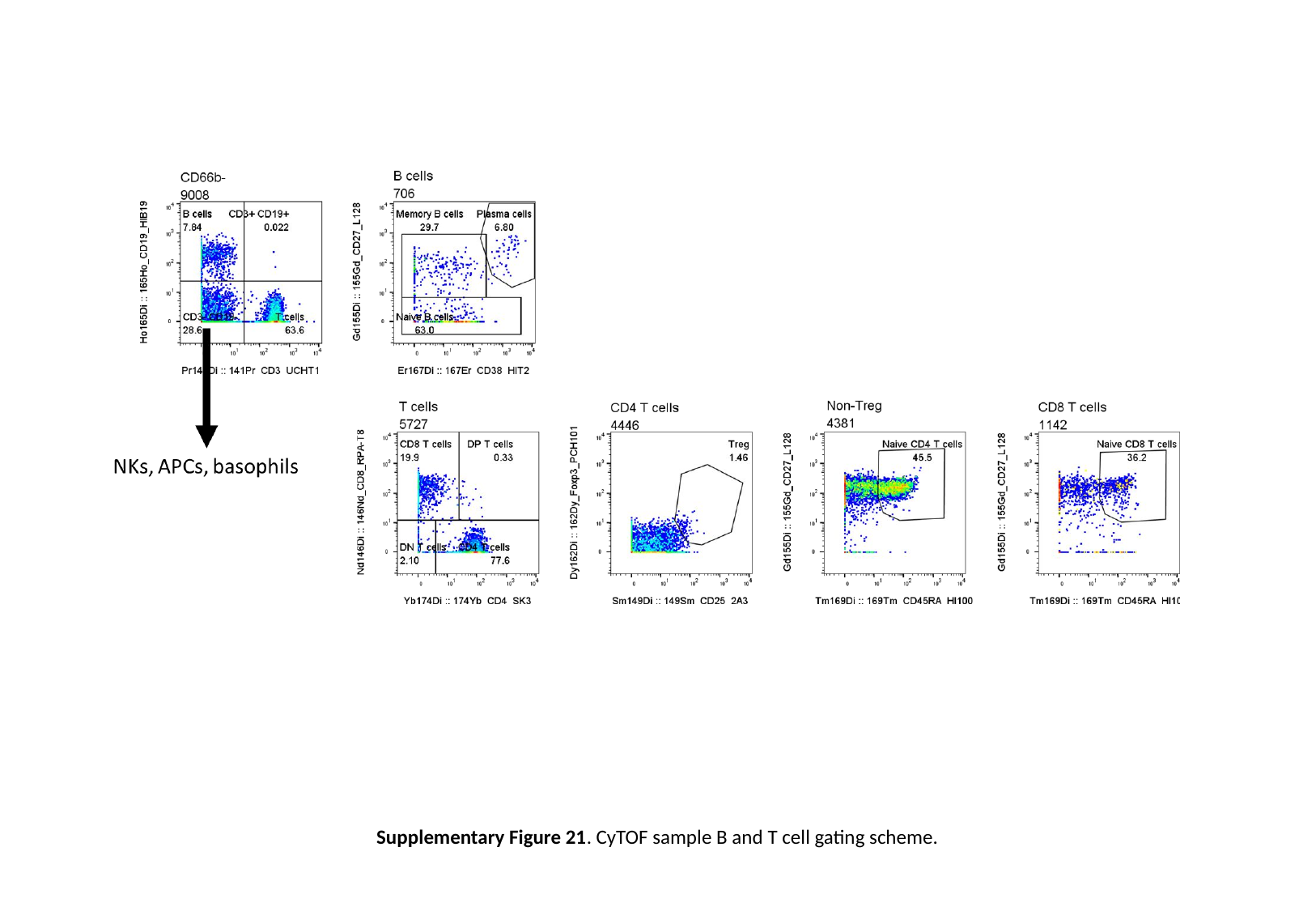

Supplementary Figure 21. CyTOF sample B and T cell gating scheme.

## Slide 22
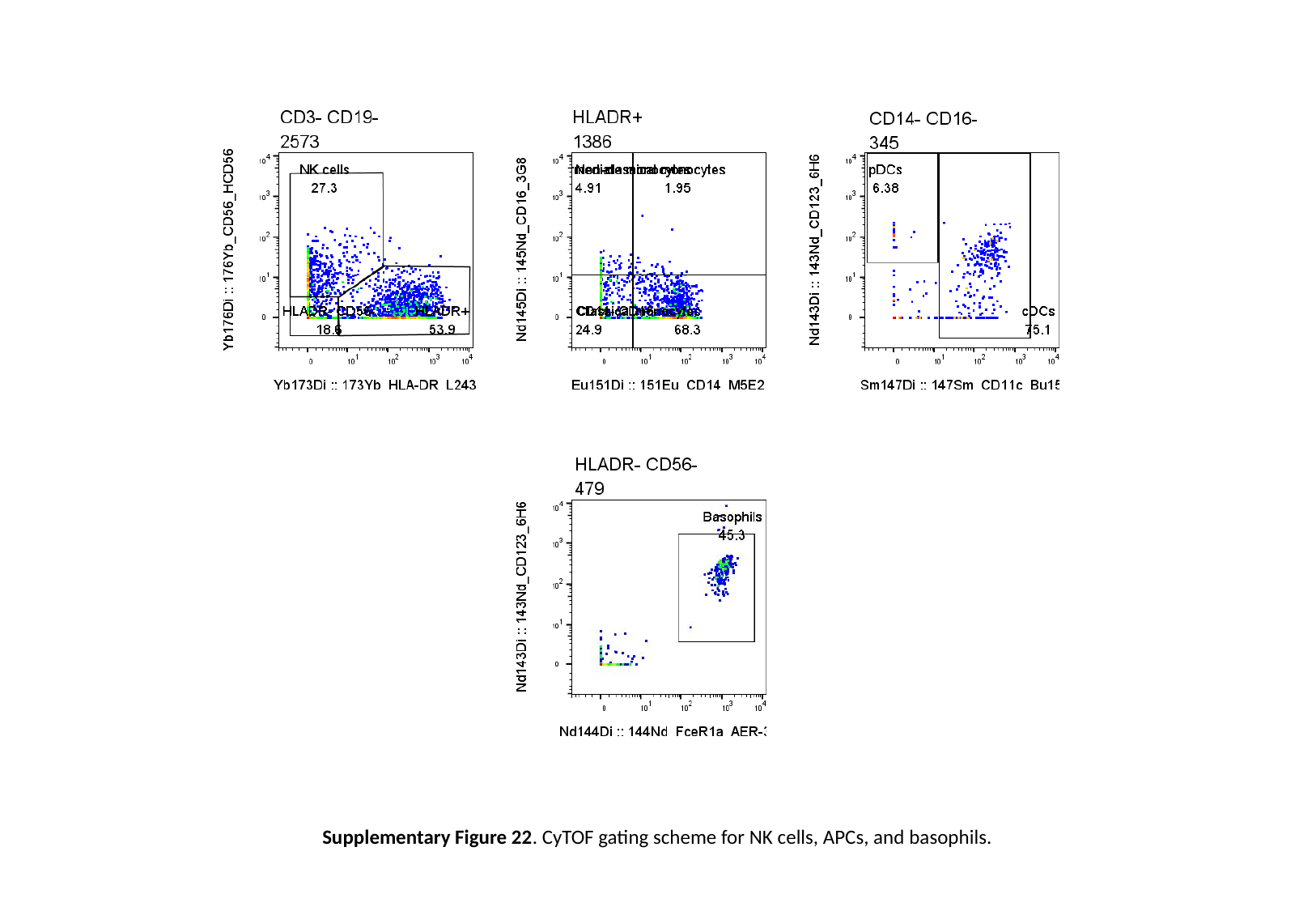

Supplementary Figure 22. CyTOF gating scheme for NK cells, APCs, and basophils.

## Slide 23
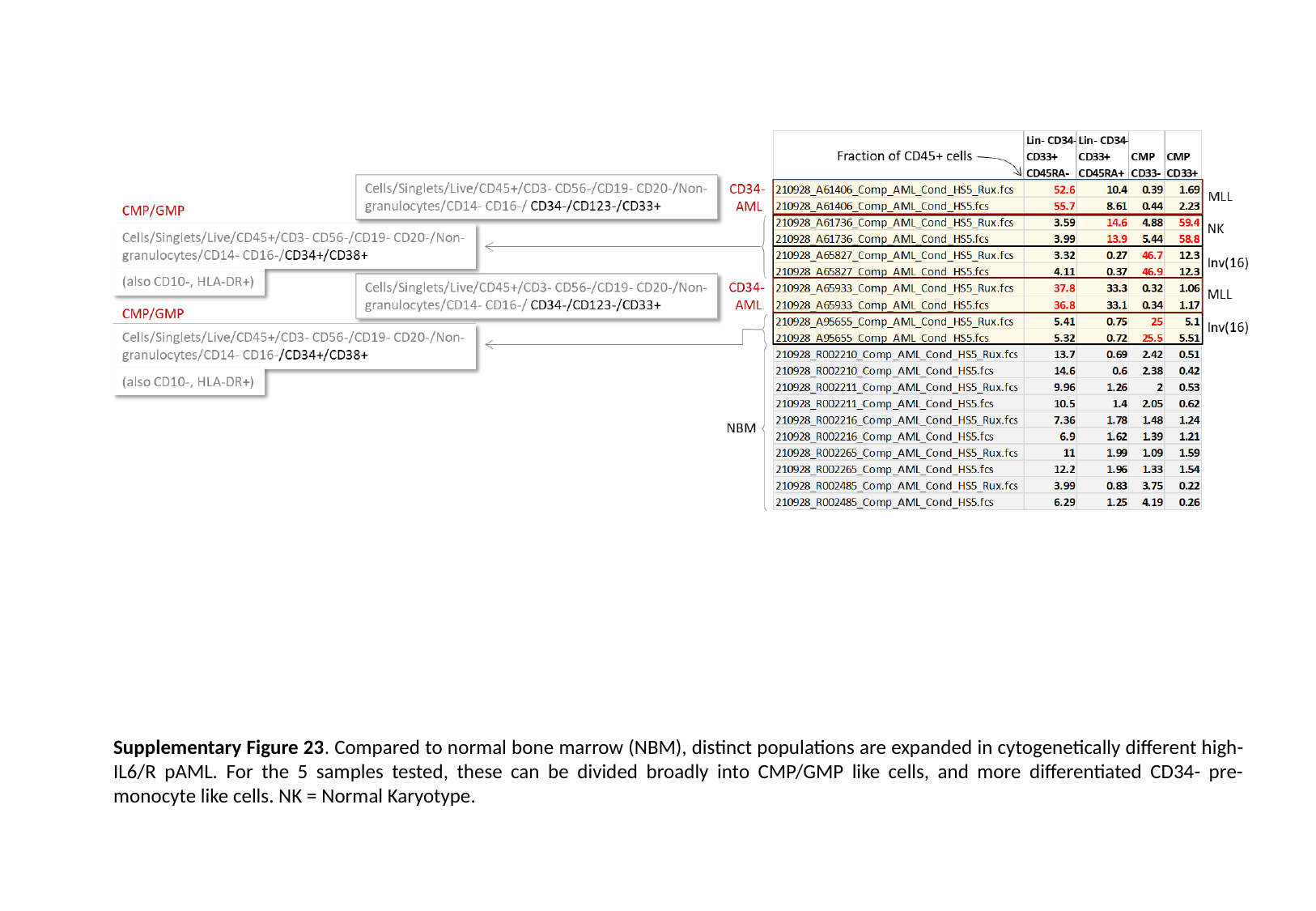

Supplementary Figure 23. Compared to normal bone marrow (NBM), distinct populations are expanded in cytogenetically different high-IL6/R pAML. For the 5 samples tested, these can be divided broadly into CMP/GMP like cells, and more differentiated CD34- pre-monocyte like cells. NK = Normal Karyotype.

## Slide 24
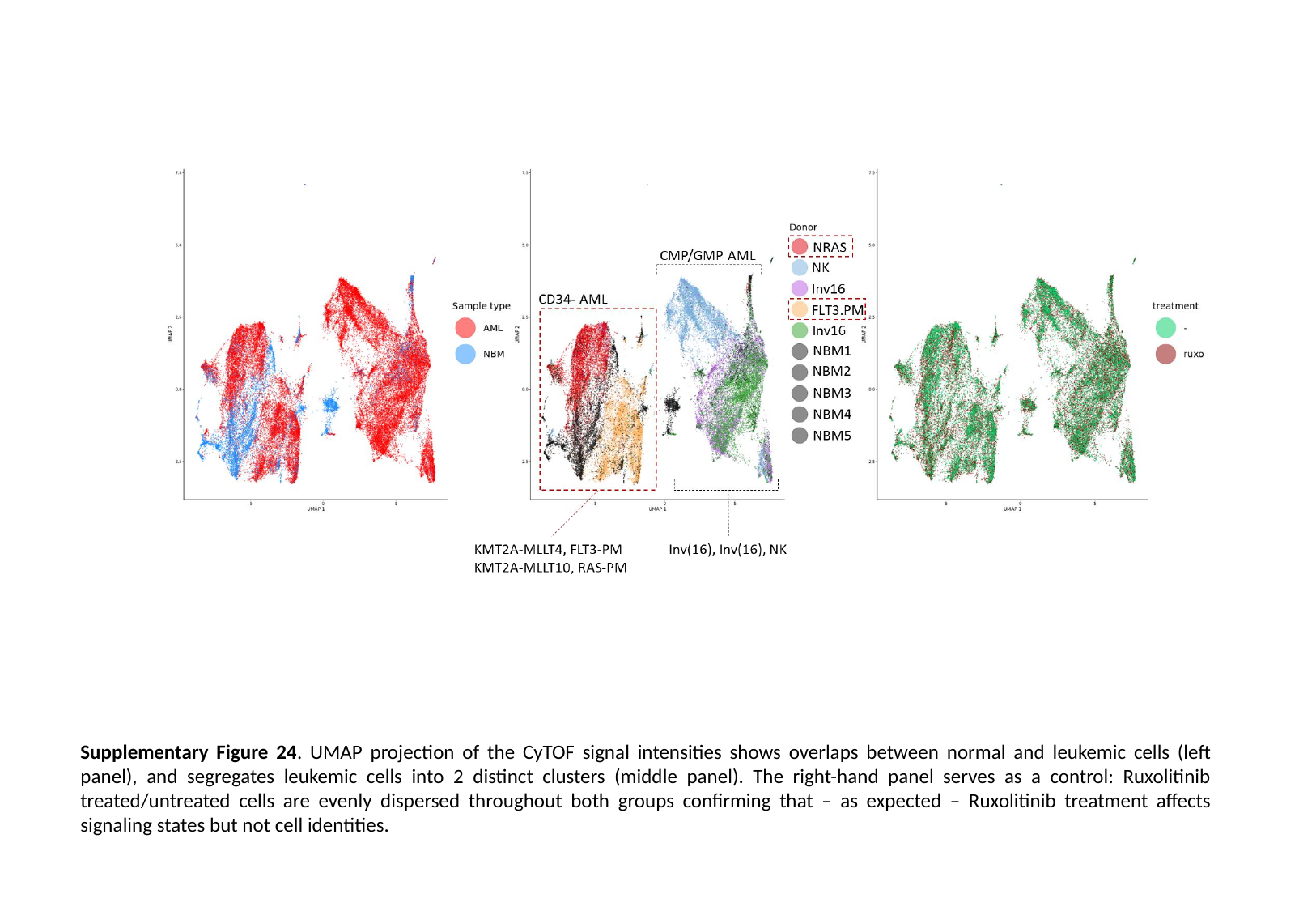

Supplementary Figure 24. UMAP projection of the CyTOF signal intensities shows overlaps between normal and leukemic cells (left panel), and segregates leukemic cells into 2 distinct clusters (middle panel). The right-hand panel serves as a control: Ruxolitinib treated/untreated cells are evenly dispersed throughout both groups confirming that – as expected – Ruxolitinib treatment affects signaling states but not cell identities.

## Slide 25
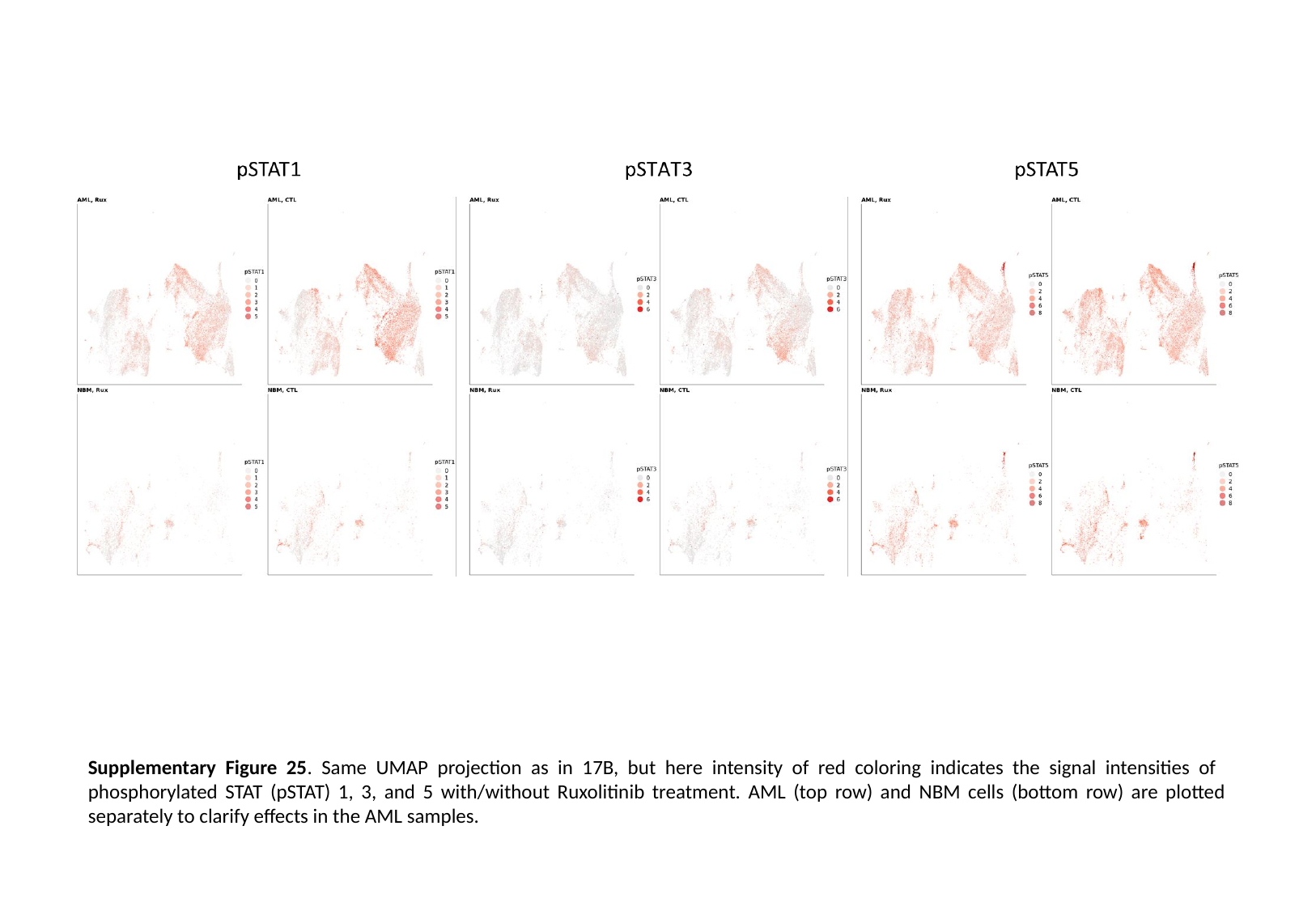

Supplementary Figure 25. Same UMAP projection as in 17B, but here intensity of red coloring indicates the signal intensities of phosphorylated STAT (pSTAT) 1, 3, and 5 with/without Ruxolitinib treatment. AML (top row) and NBM cells (bottom row) are plotted separately to clarify effects in the AML samples.

## Slide 26
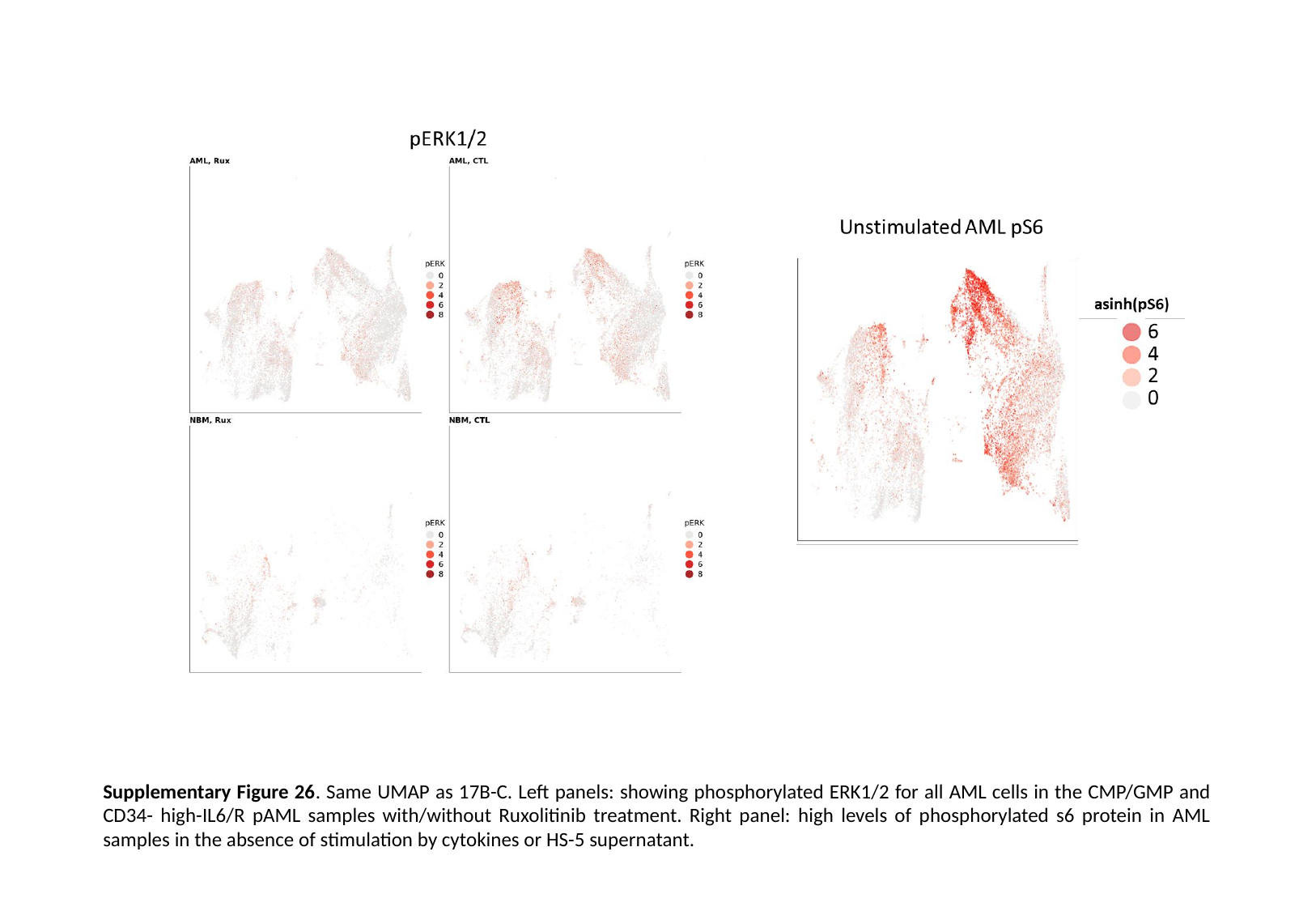

Supplementary Figure 26. Same UMAP as 17B-C. Left panels: showing phosphorylated ERK1/2 for all AML cells in the CMP/GMP and CD34- high-IL6/R pAML samples with/without Ruxolitinib treatment. Right panel: high levels of phosphorylated s6 protein in AML samples in the absence of stimulation by cytokines or HS-5 supernatant.

## Slide 27
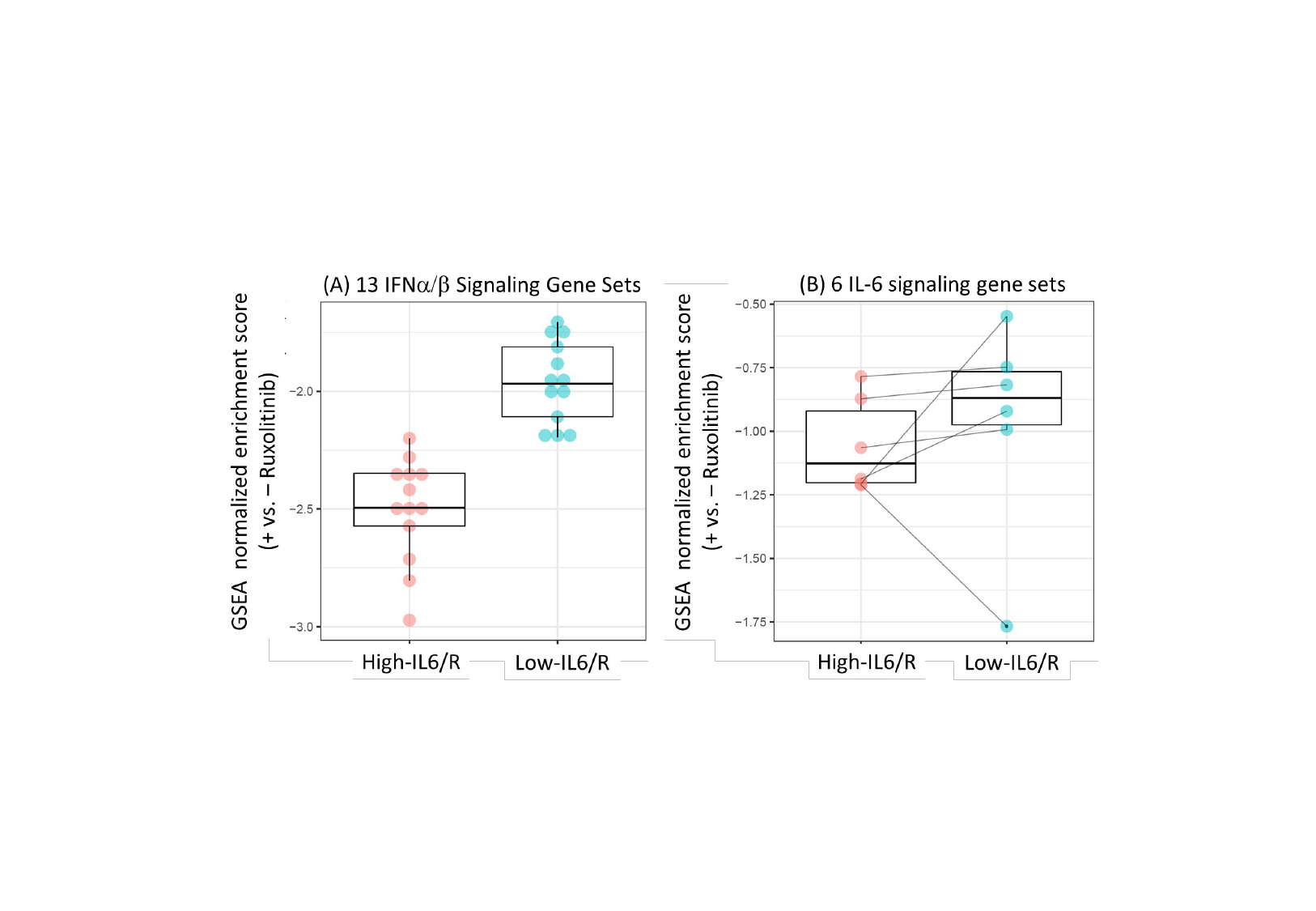

## Slide 28
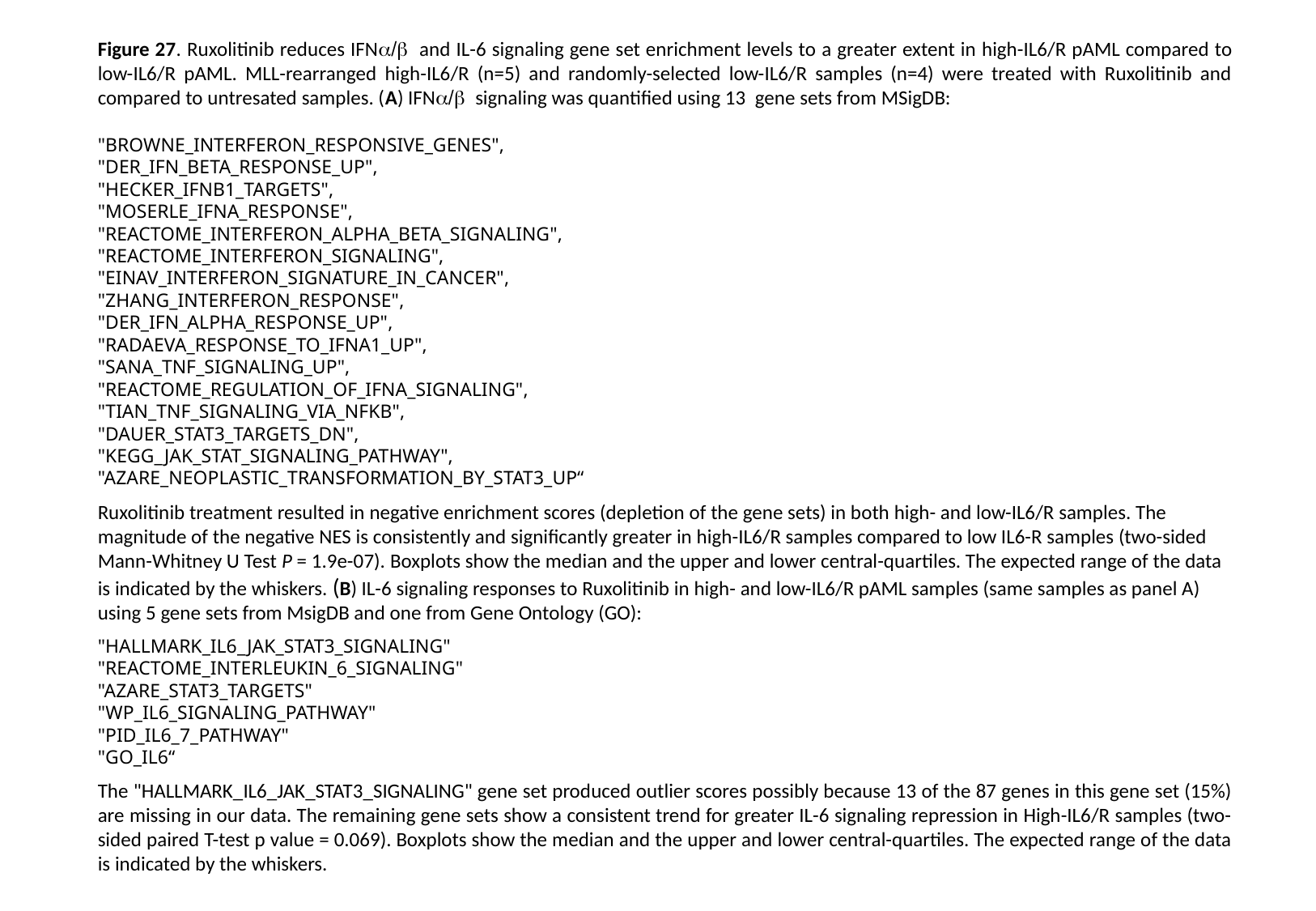

Figure 27. Ruxolitinib reduces IFNa/b and IL-6 signaling gene set enrichment levels to a greater extent in high-IL6/R pAML compared to low-IL6/R pAML. MLL-rearranged high-IL6/R (n=5) and randomly-selected low-IL6/R samples (n=4) were treated with Ruxolitinib and compared to untresated samples. (A) IFNa/b signaling was quantified using 13 gene sets from MSigDB:
"BROWNE_INTERFERON_RESPONSIVE_GENES",
"DER_IFN_BETA_RESPONSE_UP",
"HECKER_IFNB1_TARGETS",
"MOSERLE_IFNA_RESPONSE",
"REACTOME_INTERFERON_ALPHA_BETA_SIGNALING",
"REACTOME_INTERFERON_SIGNALING",
"EINAV_INTERFERON_SIGNATURE_IN_CANCER",
"ZHANG_INTERFERON_RESPONSE",
"DER_IFN_ALPHA_RESPONSE_UP",
"RADAEVA_RESPONSE_TO_IFNA1_UP",
"SANA_TNF_SIGNALING_UP",
"REACTOME_REGULATION_OF_IFNA_SIGNALING",
"TIAN_TNF_SIGNALING_VIA_NFKB",
"DAUER_STAT3_TARGETS_DN",
"KEGG_JAK_STAT_SIGNALING_PATHWAY",
"AZARE_NEOPLASTIC_TRANSFORMATION_BY_STAT3_UP“
Ruxolitinib treatment resulted in negative enrichment scores (depletion of the gene sets) in both high- and low-IL6/R samples. The magnitude of the negative NES is consistently and significantly greater in high-IL6/R samples compared to low IL6-R samples (two-sided Mann-Whitney U Test P = 1.9e-07). Boxplots show the median and the upper and lower central-quartiles. The expected range of the data is indicated by the whiskers. (B) IL-6 signaling responses to Ruxolitinib in high- and low-IL6/R pAML samples (same samples as panel A) using 5 gene sets from MsigDB and one from Gene Ontology (GO):
"HALLMARK_IL6_JAK_STAT3_SIGNALING"
"REACTOME_INTERLEUKIN_6_SIGNALING"
"AZARE_STAT3_TARGETS"
"WP_IL6_SIGNALING_PATHWAY"
"PID_IL6_7_PATHWAY"
"GO_IL6“
The "HALLMARK_IL6_JAK_STAT3_SIGNALING" gene set produced outlier scores possibly because 13 of the 87 genes in this gene set (15%) are missing in our data. The remaining gene sets show a consistent trend for greater IL-6 signaling repression in High-IL6/R samples (two-sided paired T-test p value = 0.069). Boxplots show the median and the upper and lower central-quartiles. The expected range of the data is indicated by the whiskers.

## Slide 29
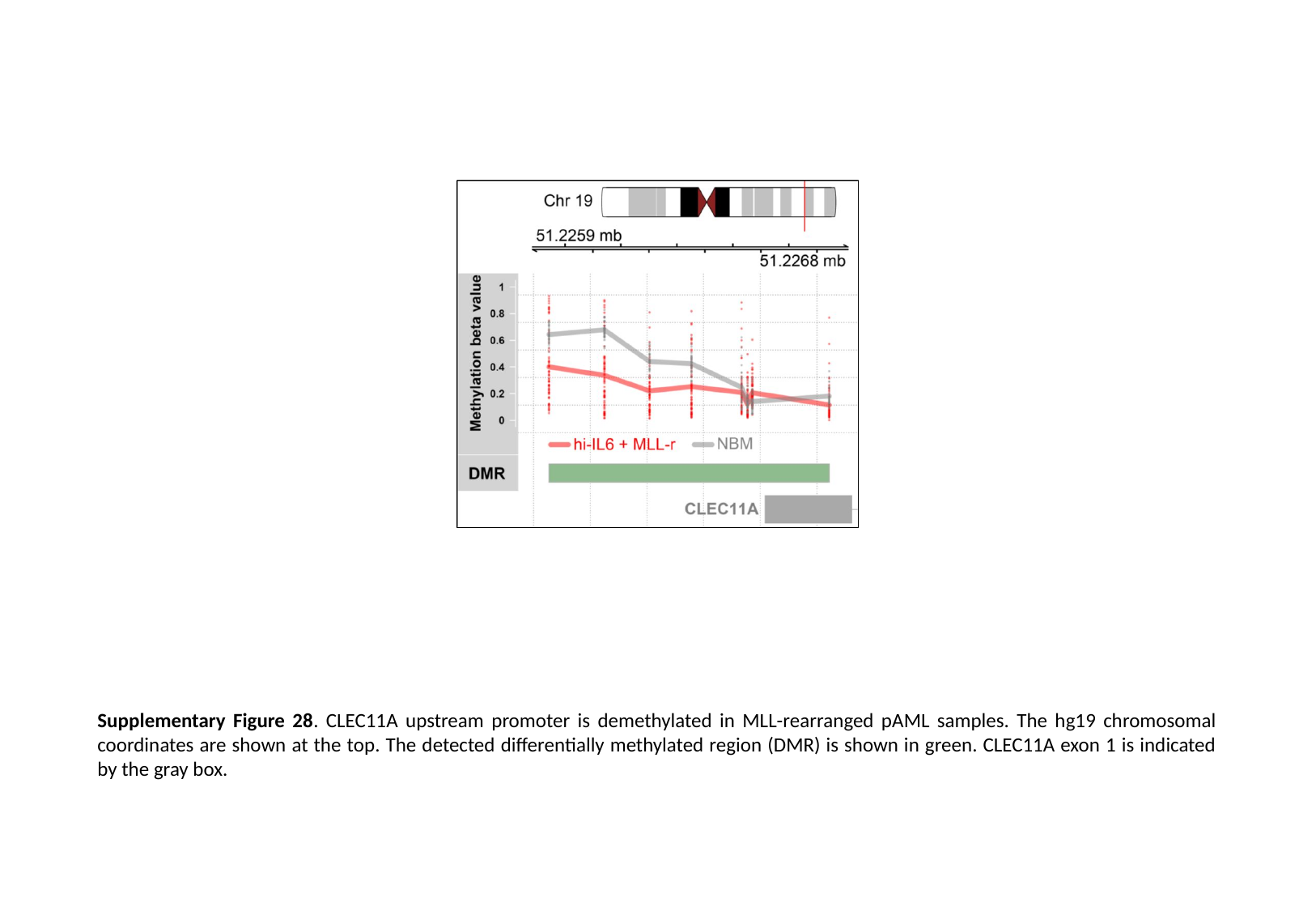

Supplementary Figure 28. CLEC11A upstream promoter is demethylated in MLL-rearranged pAML samples. The hg19 chromosomal coordinates are shown at the top. The detected differentially methylated region (DMR) is shown in green. CLEC11A exon 1 is indicated by the gray box.

## Slide 30
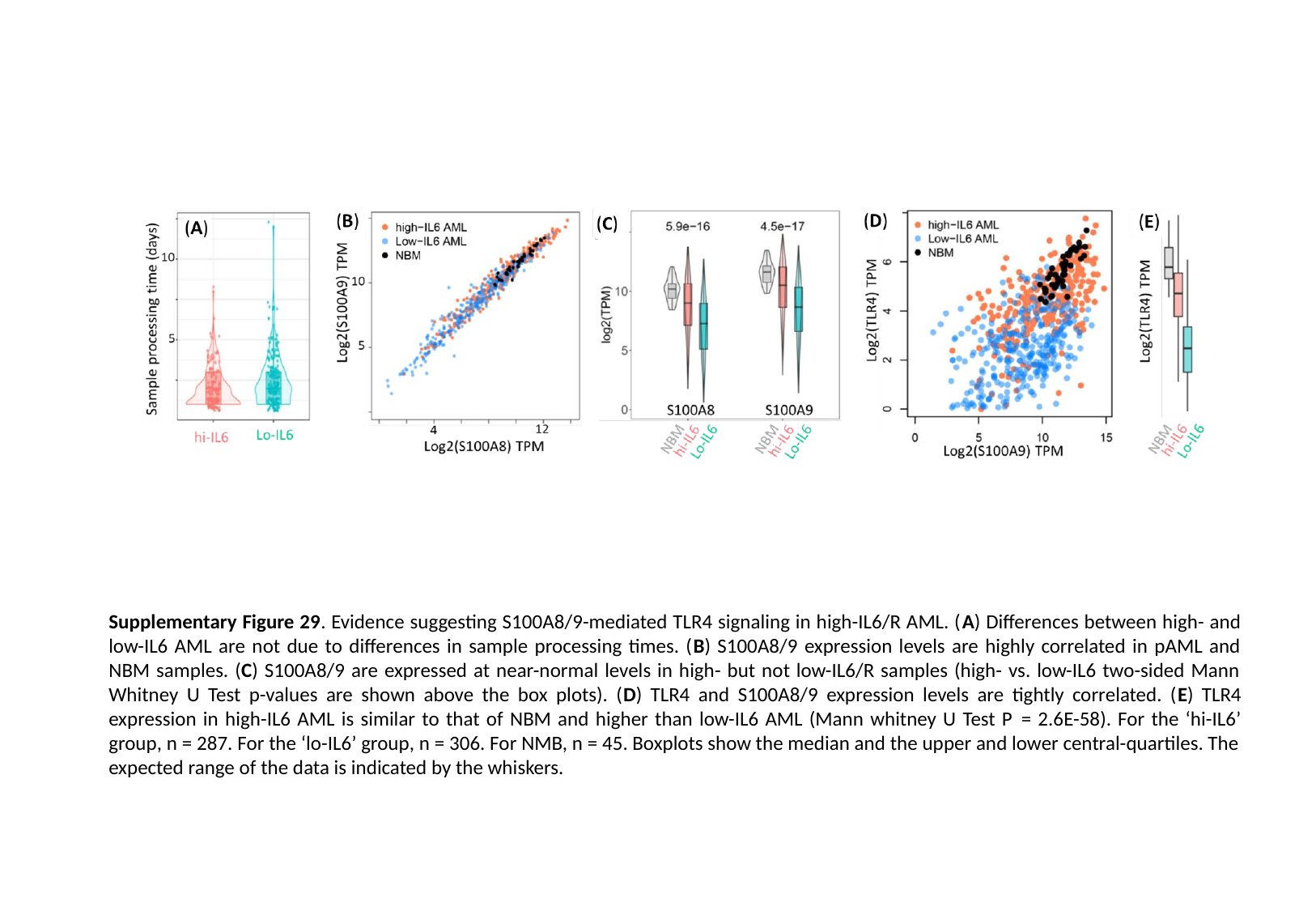

Supplementary Figure 29. Evidence suggesting S100A8/9-mediated TLR4 signaling in high-IL6/R AML. (A) Differences between high- and low-IL6 AML are not due to differences in sample processing times. (B) S100A8/9 expression levels are highly correlated in pAML and NBM samples. (C) S100A8/9 are expressed at near-normal levels in high- but not low-IL6/R samples (high- vs. low-IL6 two-sided Mann Whitney U Test p-values are shown above the box plots). (D) TLR4 and S100A8/9 expression levels are tightly correlated. (E) TLR4 expression in high-IL6 AML is similar to that of NBM and higher than low-IL6 AML (Mann whitney U Test P = 2.6E-58). For the ‘hi-IL6’ group, n = 287. For the ‘lo-IL6’ group, n = 306. For NMB, n = 45. Boxplots show the median and the upper and lower central-quartiles. The expected range of the data is indicated by the whiskers.

## Slide 31
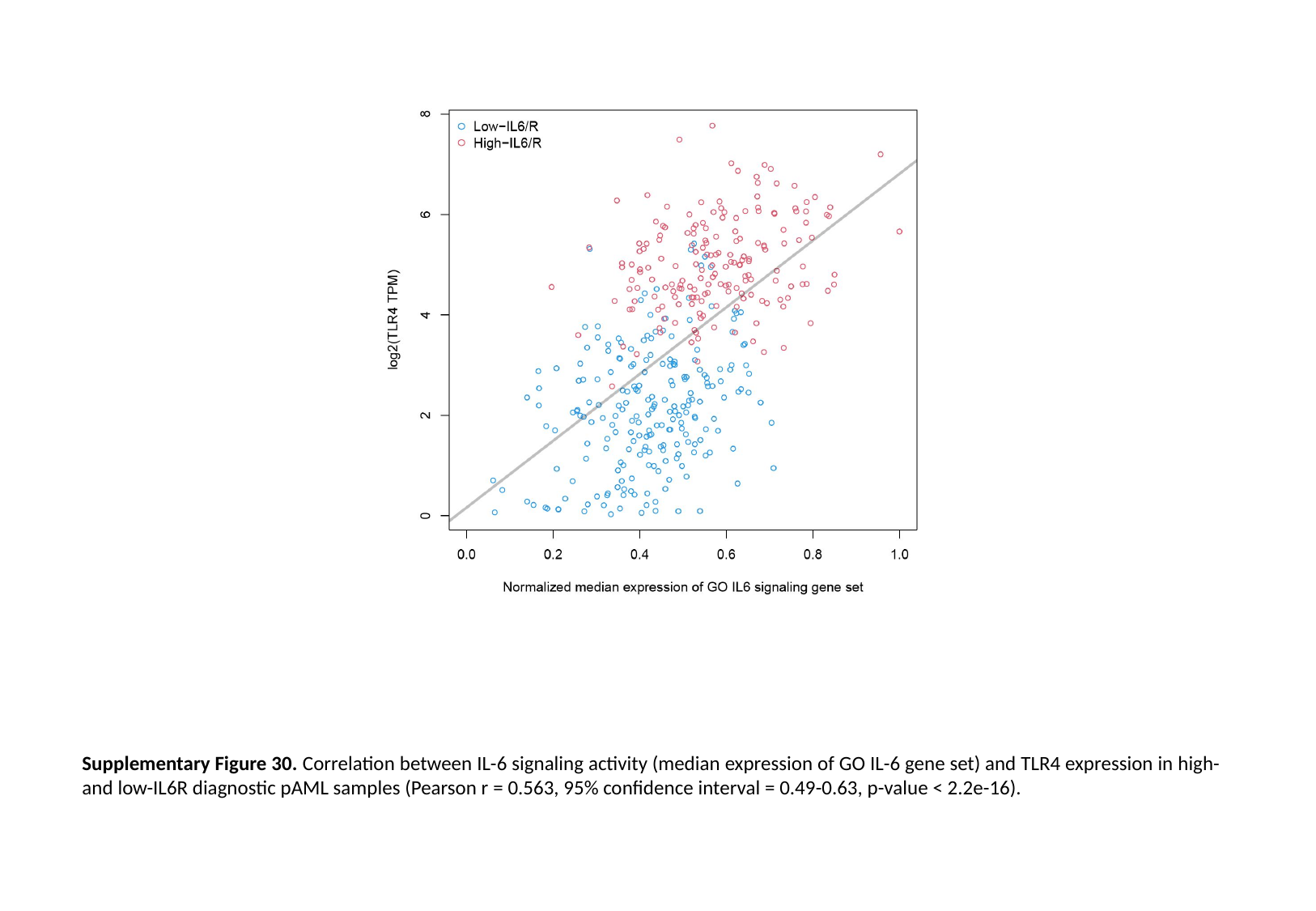

Supplementary Figure 30. Correlation between IL-6 signaling activity (median expression of GO IL-6 gene set) and TLR4 expression in high- and low-IL6R diagnostic pAML samples (Pearson r = 0.563, 95% confidence interval = 0.49-0.63, p-value < 2.2e-16).

## Slide 32
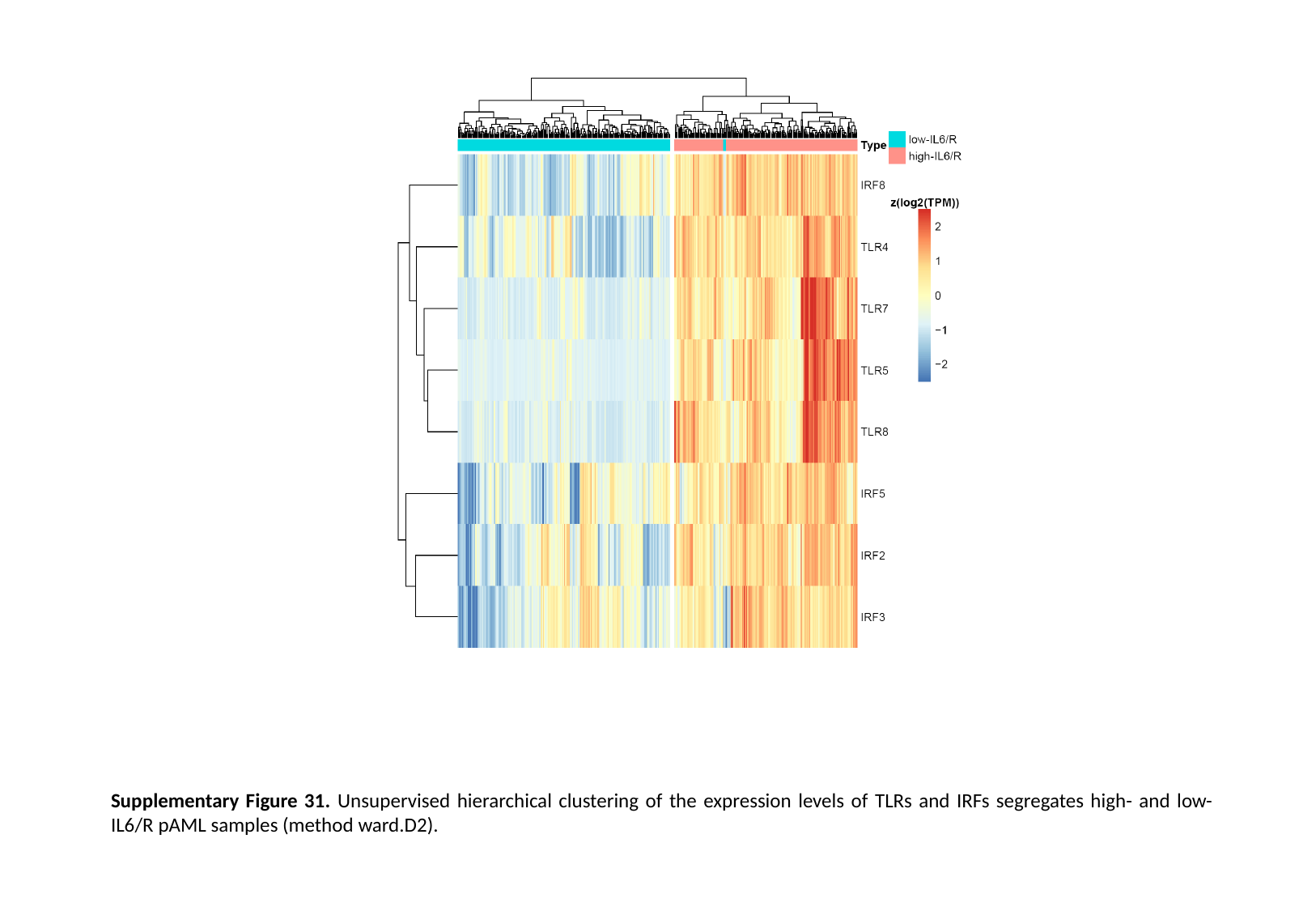

Supplementary Figure 31. Unsupervised hierarchical clustering of the expression levels of TLRs and IRFs segregates high- and low-IL6/R pAML samples (method ward.D2).

## Slide 33
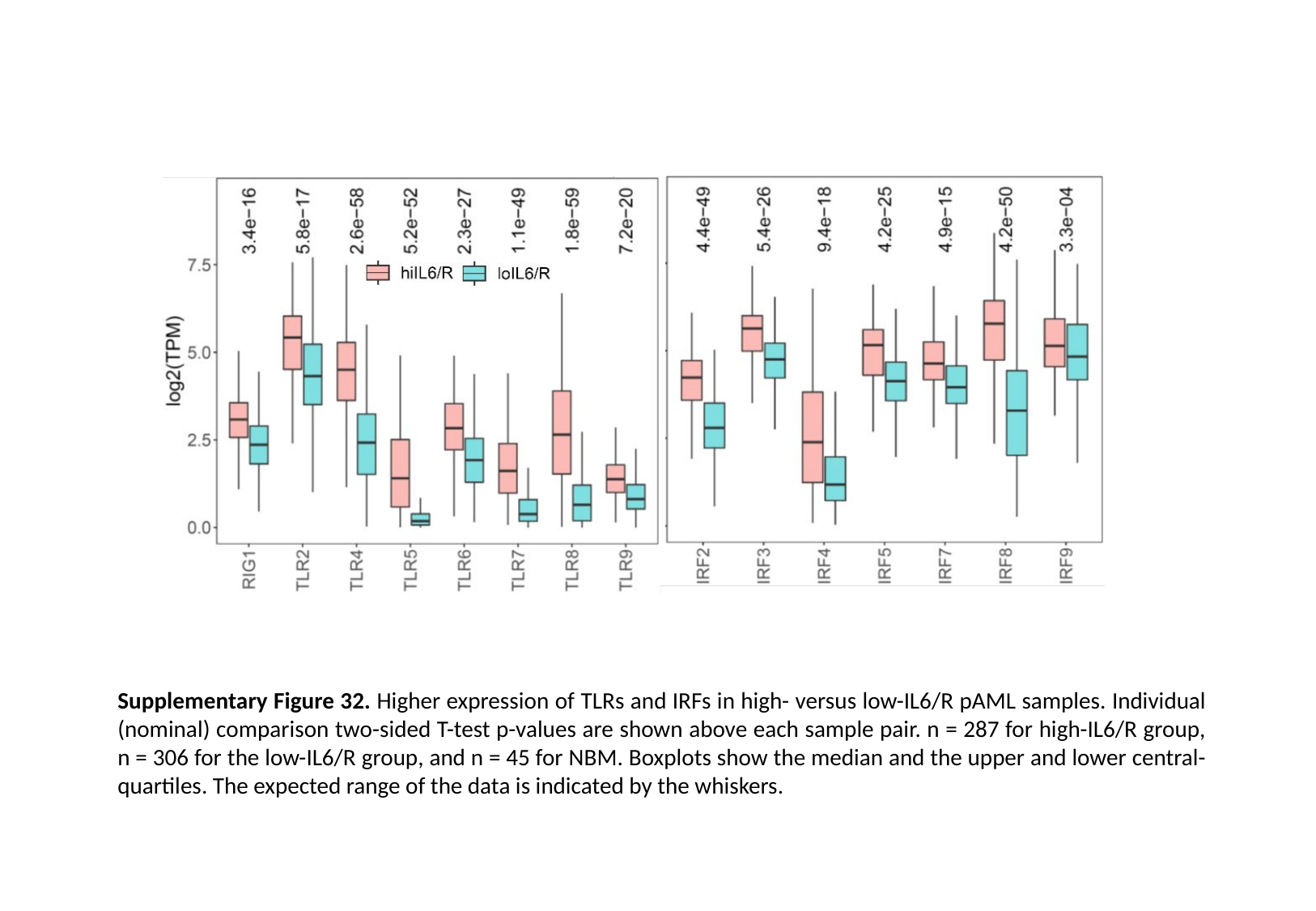

Supplementary Figure 32. Higher expression of TLRs and IRFs in high- versus low-IL6/R pAML samples. Individual (nominal) comparison two-sided T-test p-values are shown above each sample pair. n = 287 for high-IL6/R group, n = 306 for the low-IL6/R group, and n = 45 for NBM. Boxplots show the median and the upper and lower central-quartiles. The expected range of the data is indicated by the whiskers.

## Slide 34
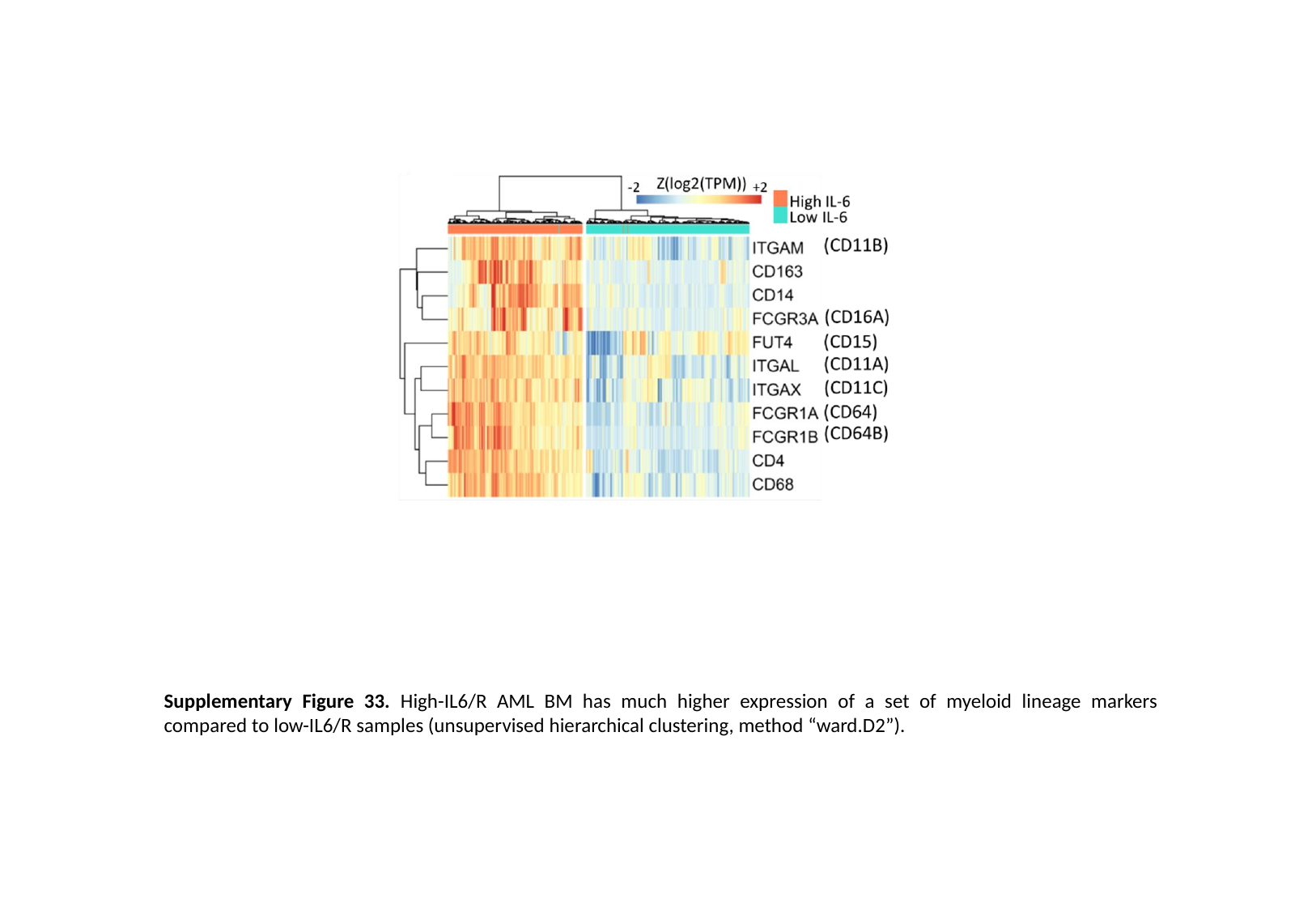

Supplementary Figure 33. High-IL6/R AML BM has much higher expression of a set of myeloid lineage markers compared to low-IL6/R samples (unsupervised hierarchical clustering, method “ward.D2”).

## Slide 35
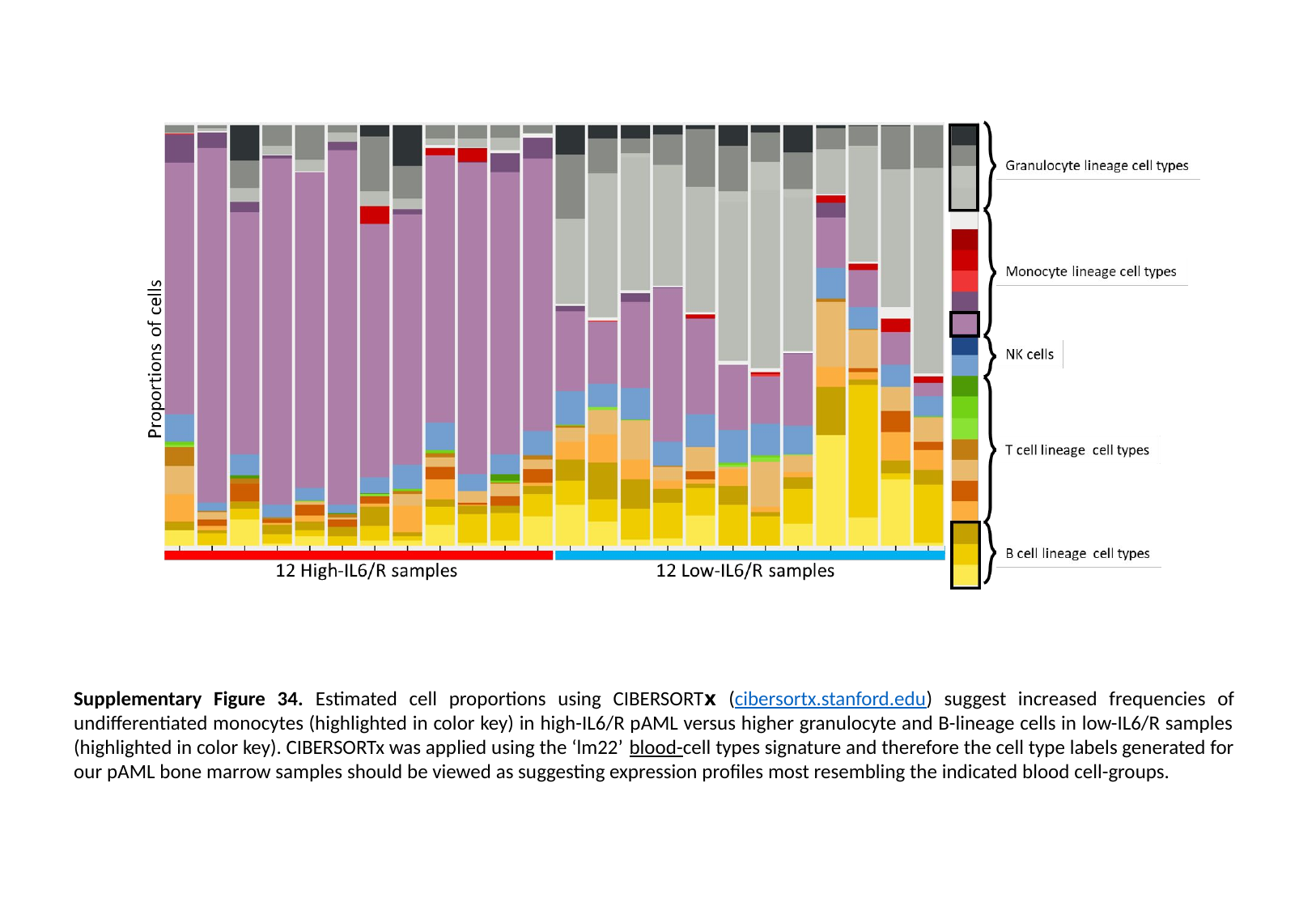

Supplementary Figure 34. Estimated cell proportions using CIBERSORTx (cibersortx.stanford.edu) suggest increased frequencies of undifferentiated monocytes (highlighted in color key) in high-IL6/R pAML versus higher granulocyte and B-lineage cells in low-IL6/R samples (highlighted in color key). CIBERSORTx was applied using the ‘lm22’ blood-cell types signature and therefore the cell type labels generated for our pAML bone marrow samples should be viewed as suggesting expression profiles most resembling the indicated blood cell-groups.

## Slide 36
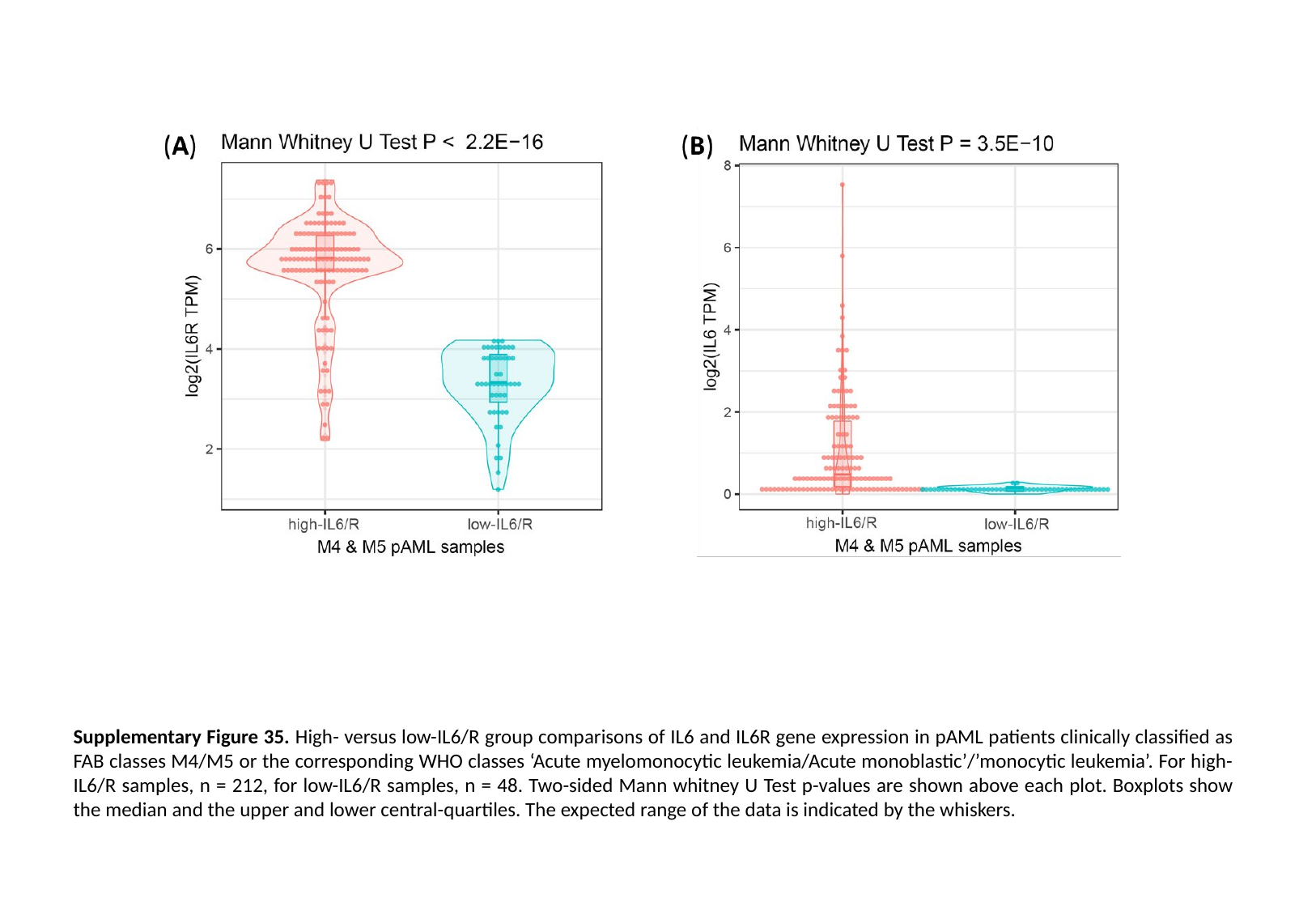

Supplementary Figure 35. High- versus low-IL6/R group comparisons of IL6 and IL6R gene expression in pAML patients clinically classified as FAB classes M4/M5 or the corresponding WHO classes ‘Acute myelomonocytic leukemia/Acute monoblastic’/’monocytic leukemia’. For high-IL6/R samples, n = 212, for low-IL6/R samples, n = 48. Two-sided Mann whitney U Test p-values are shown above each plot. Boxplots show the median and the upper and lower central-quartiles. The expected range of the data is indicated by the whiskers.

## Slide 37
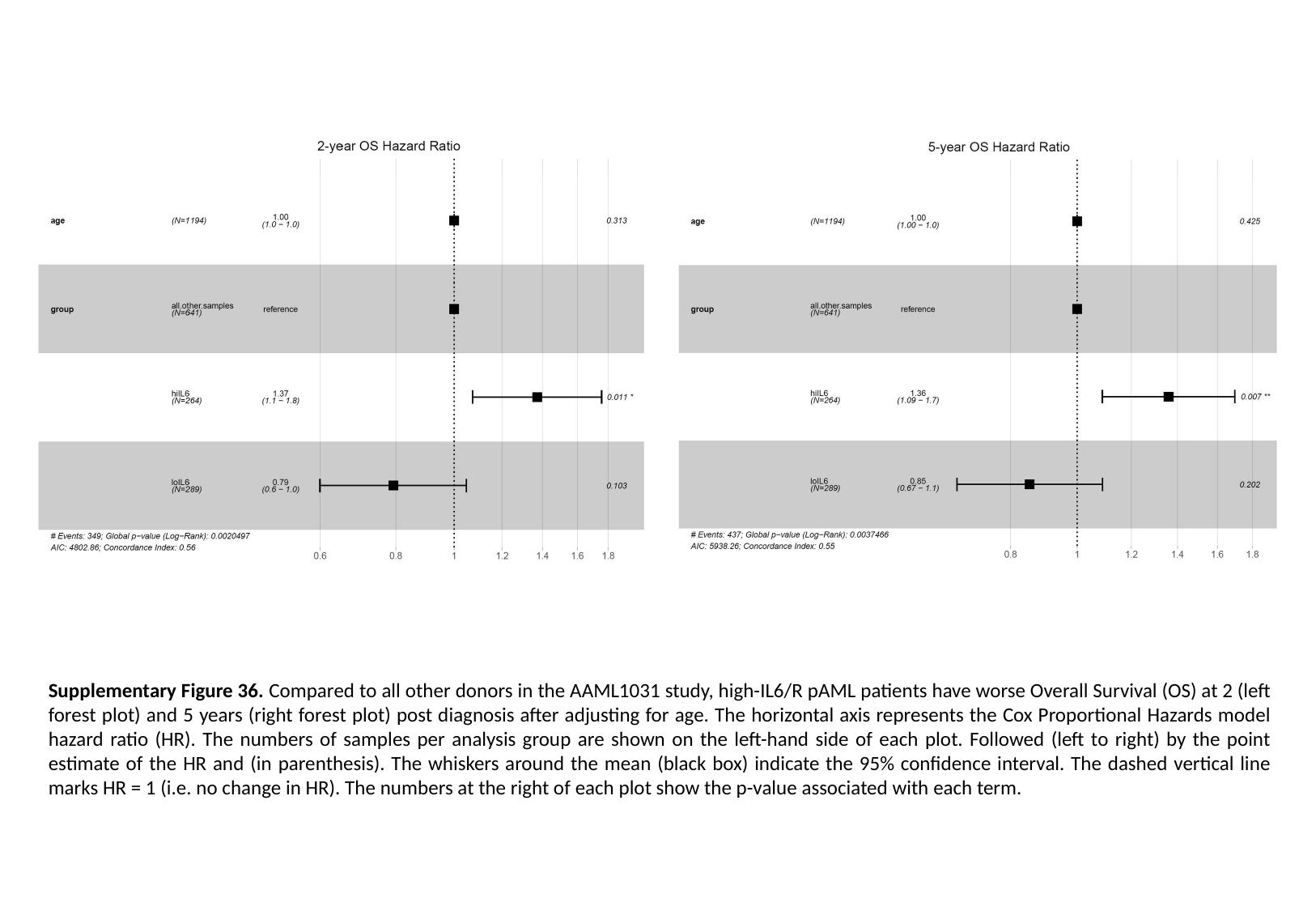

Supplementary Figure 36. Compared to all other donors in the AAML1031 study, high-IL6/R pAML patients have worse Overall Survival (OS) at 2 (left forest plot) and 5 years (right forest plot) post diagnosis after adjusting for age. The horizontal axis represents the Cox Proportional Hazards model hazard ratio (HR). The numbers of samples per analysis group are shown on the left-hand side of each plot. Followed (left to right) by the point estimate of the HR and (in parenthesis). The whiskers around the mean (black box) indicate the 95% confidence interval. The dashed vertical line marks HR = 1 (i.e. no change in HR). The numbers at the right of each plot show the p-value associated with each term.

## Slide 38
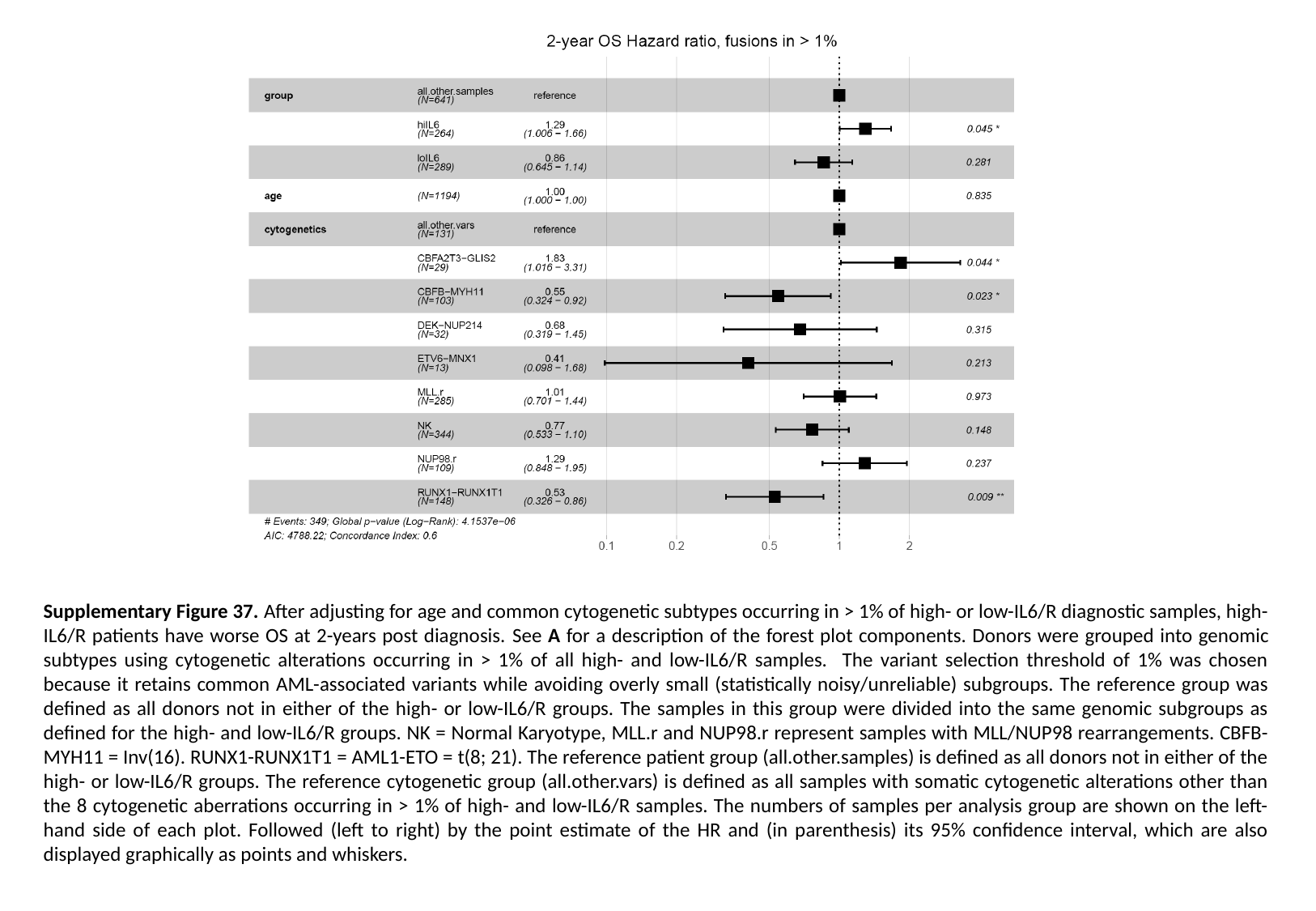

Supplementary Figure 37. After adjusting for age and common cytogenetic subtypes occurring in > 1% of high- or low-IL6/R diagnostic samples, high-IL6/R patients have worse OS at 2-years post diagnosis. See A for a description of the forest plot components. Donors were grouped into genomic subtypes using cytogenetic alterations occurring in > 1% of all high- and low-IL6/R samples. The variant selection threshold of 1% was chosen because it retains common AML-associated variants while avoiding overly small (statistically noisy/unreliable) subgroups. The reference group was defined as all donors not in either of the high- or low-IL6/R groups. The samples in this group were divided into the same genomic subgroups as defined for the high- and low-IL6/R groups. NK = Normal Karyotype, MLL.r and NUP98.r represent samples with MLL/NUP98 rearrangements. CBFB-MYH11 = Inv(16). RUNX1-RUNX1T1 = AML1-ETO = t(8; 21). The reference patient group (all.other.samples) is defined as all donors not in either of the high- or low-IL6/R groups. The reference cytogenetic group (all.other.vars) is defined as all samples with somatic cytogenetic alterations other than the 8 cytogenetic aberrations occurring in > 1% of high- and low-IL6/R samples. The numbers of samples per analysis group are shown on the left-hand side of each plot. Followed (left to right) by the point estimate of the HR and (in parenthesis) its 95% confidence interval, which are also displayed graphically as points and whiskers.

## Slide 39
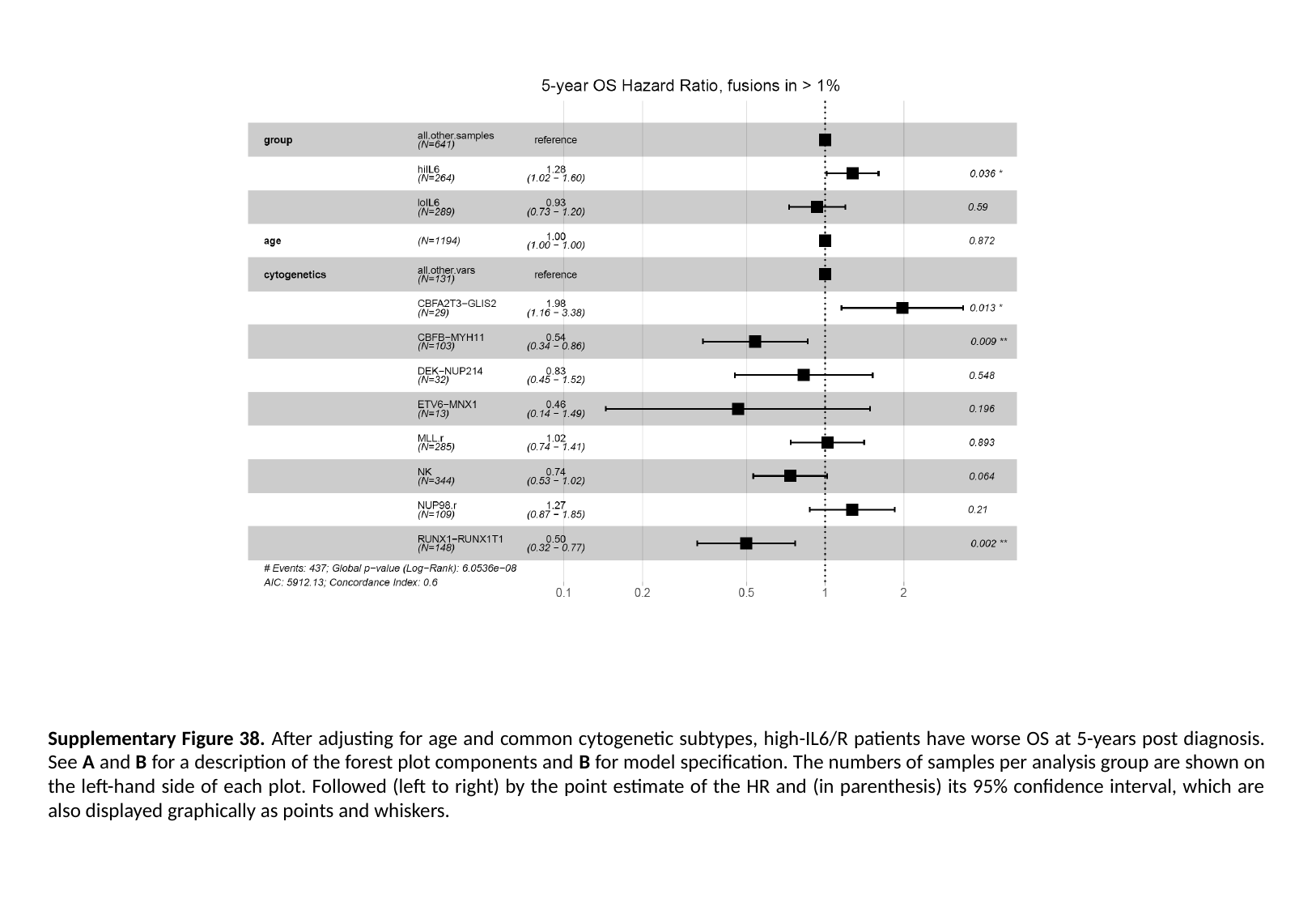

Supplementary Figure 38. After adjusting for age and common cytogenetic subtypes, high-IL6/R patients have worse OS at 5-years post diagnosis. See A and B for a description of the forest plot components and B for model specification. The numbers of samples per analysis group are shown on the left-hand side of each plot. Followed (left to right) by the point estimate of the HR and (in parenthesis) its 95% confidence interval, which are also displayed graphically as points and whiskers.

## Slide 40
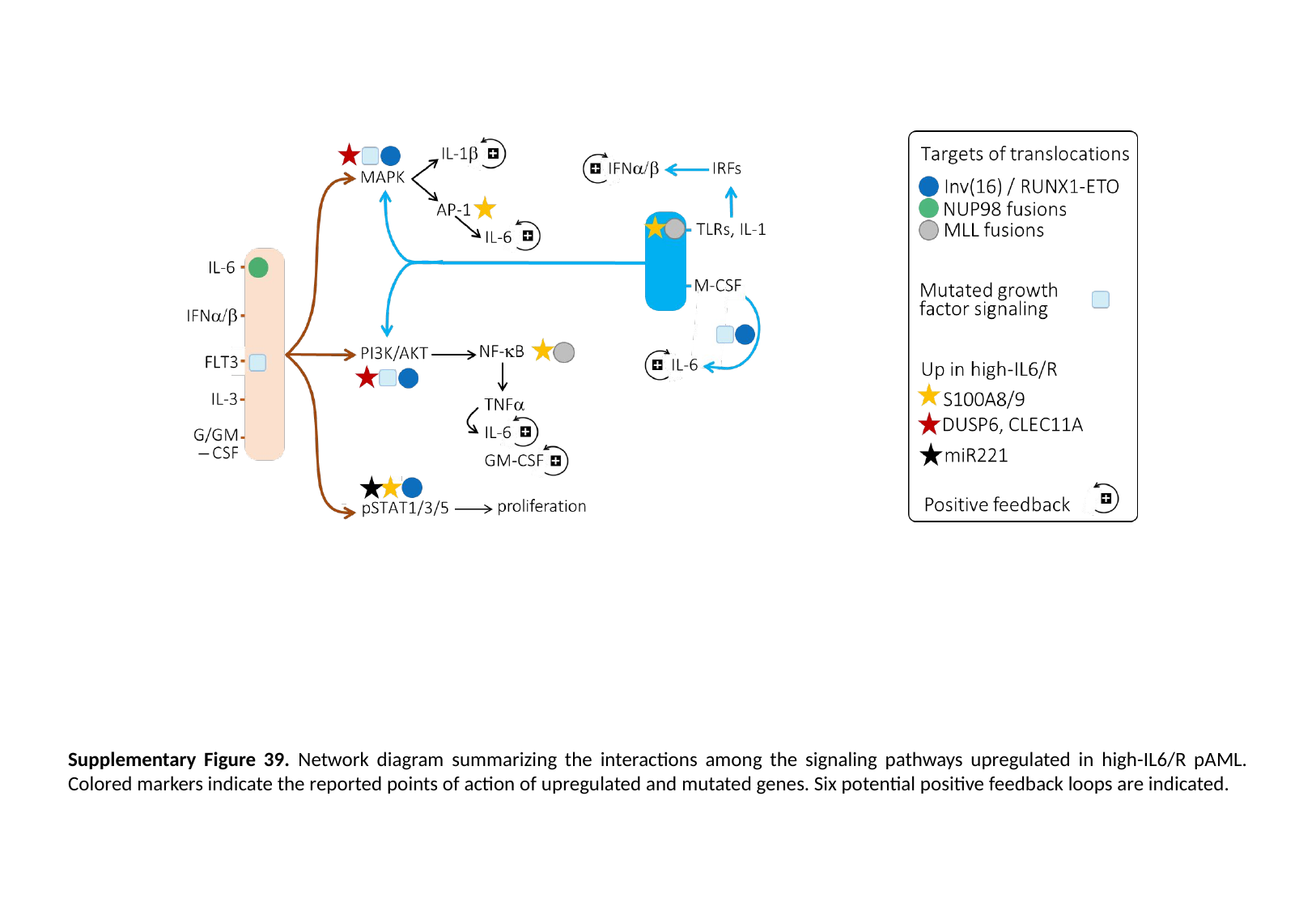

Supplementary Figure 39. Network diagram summarizing the interactions among the signaling pathways upregulated in high-IL6/R pAML. Colored markers indicate the reported points of action of upregulated and mutated genes. Six potential positive feedback loops are indicated.

## Slide 41
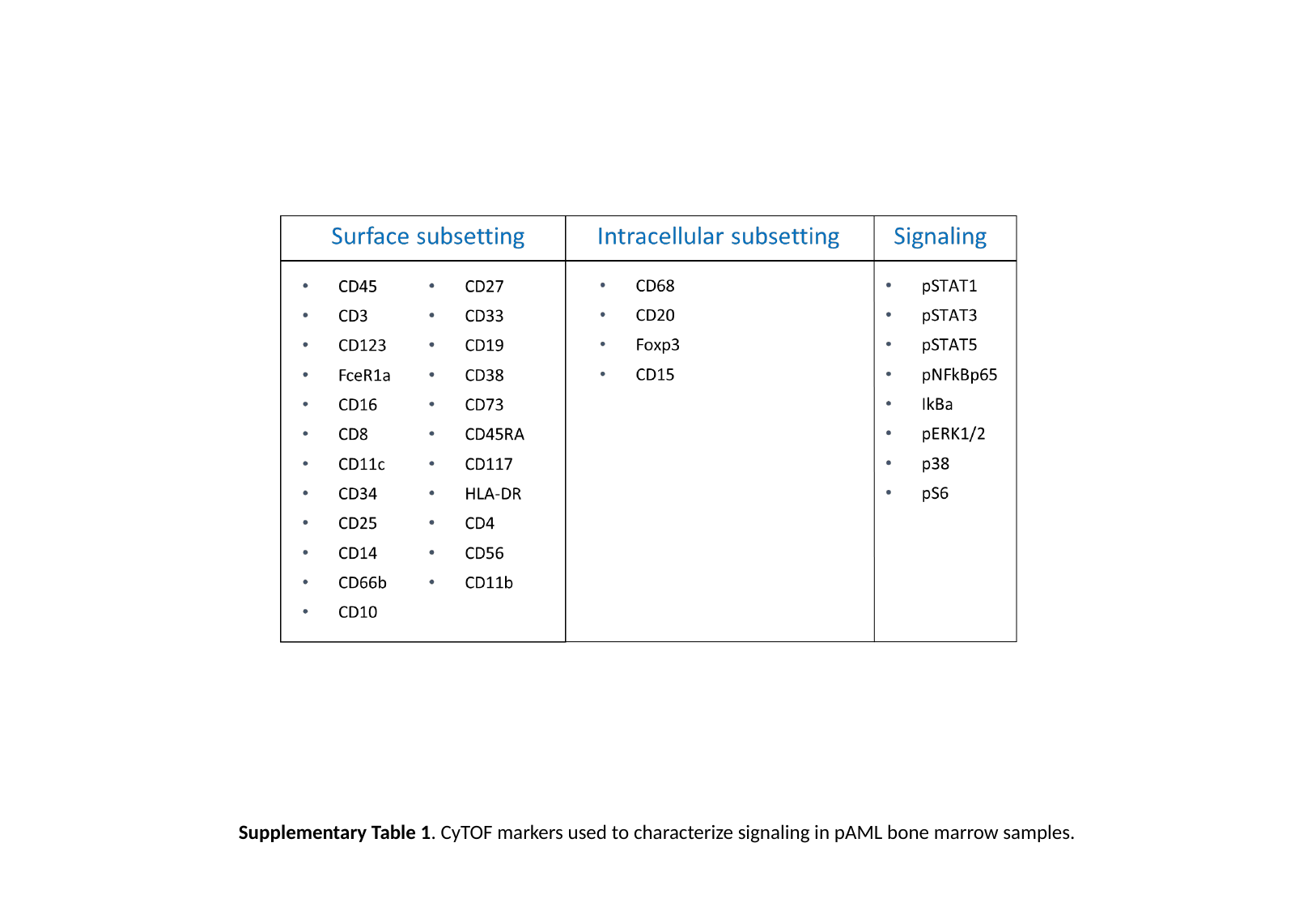

Supplementary Table 1. CyTOF markers used to characterize signaling in pAML bone marrow samples.

## Slide 42
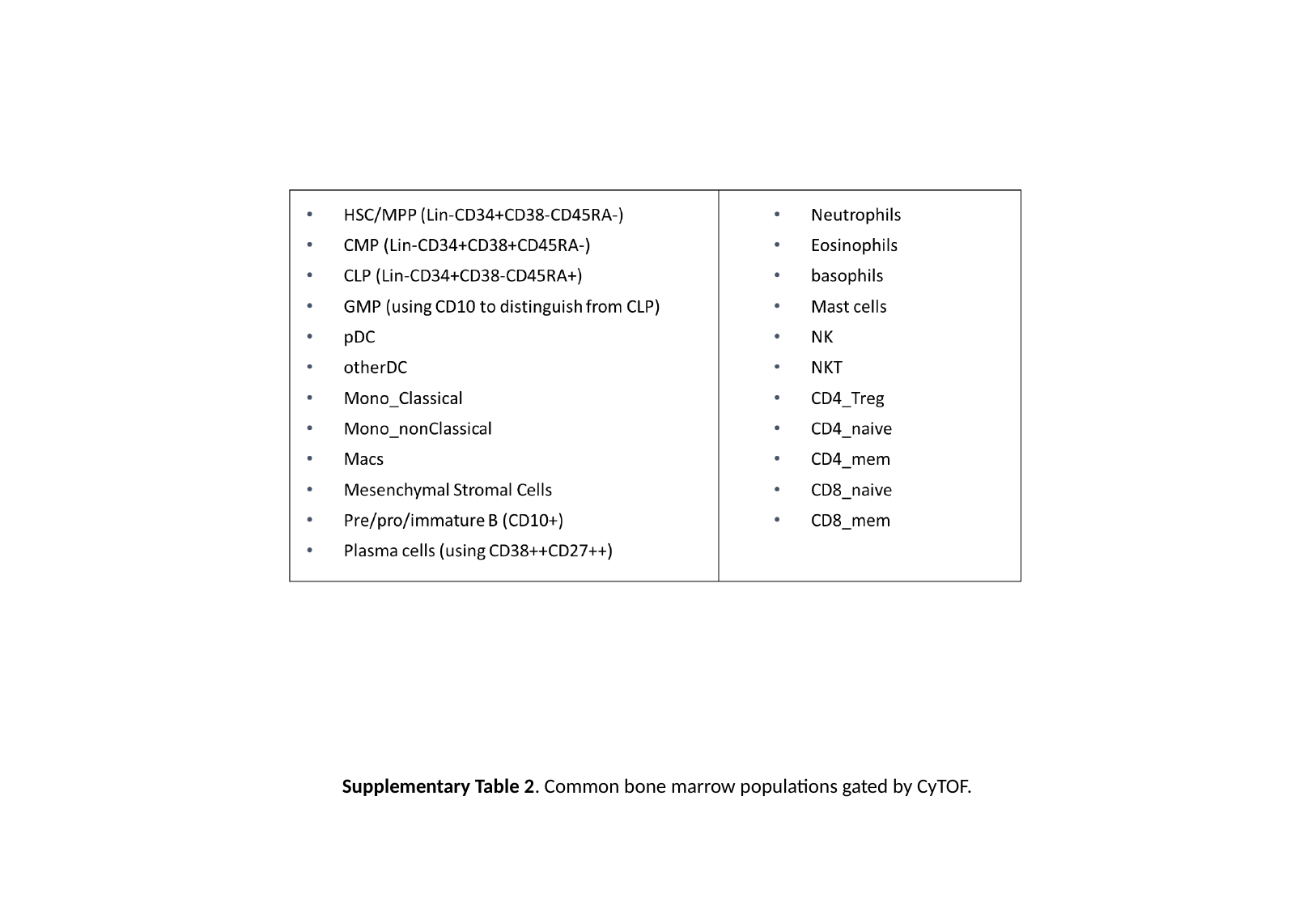

Supplementary Table 2. Common bone marrow populations gated by CyTOF.

## Slide 43
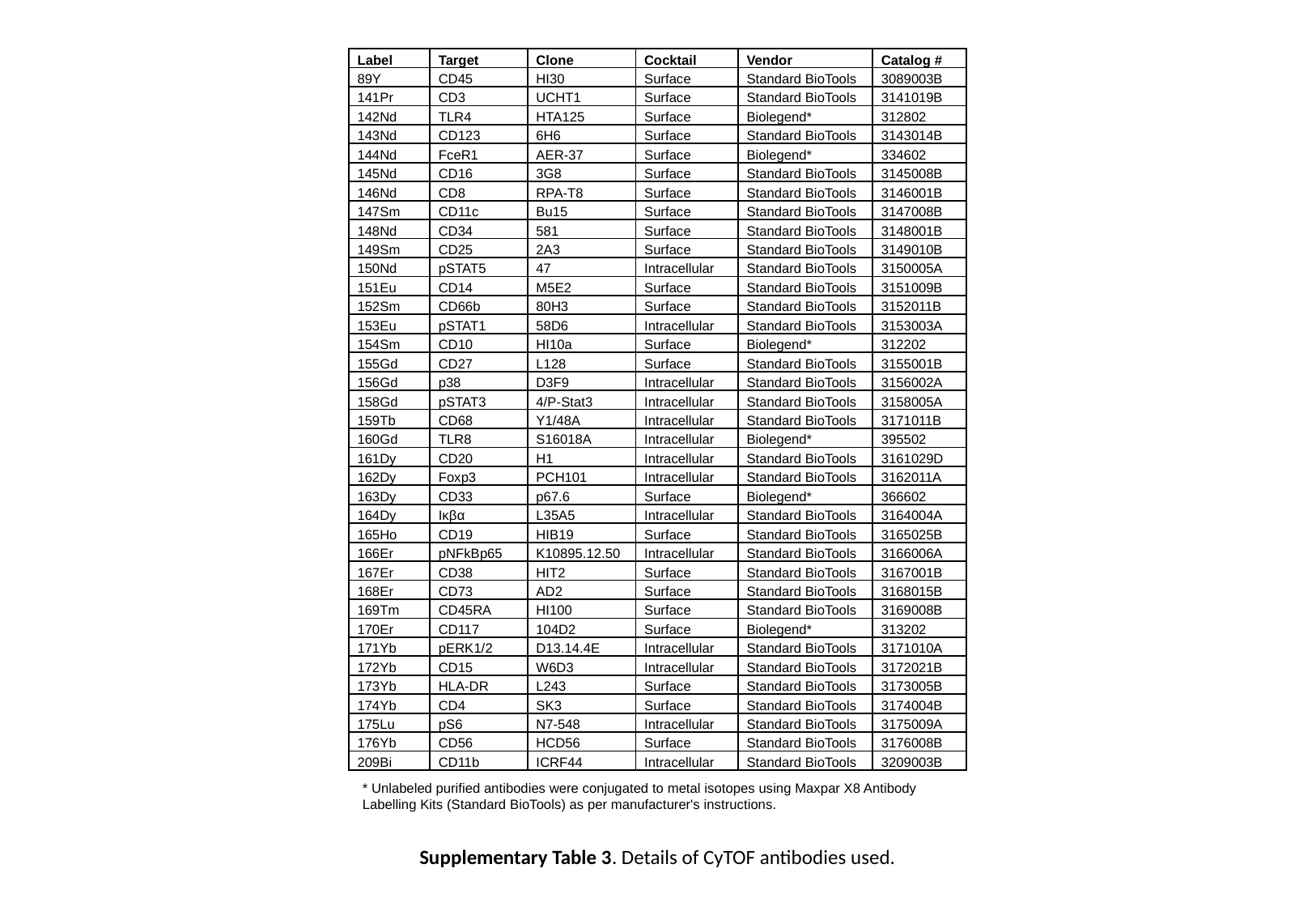

| Label | Target | Clone | Cocktail | Vendor | Catalog # |
| --- | --- | --- | --- | --- | --- |
| 89Y | CD45 | HI30 | Surface | Standard BioTools | 3089003B |
| 141Pr | CD3 | UCHT1 | Surface | Standard BioTools | 3141019B |
| 142Nd | TLR4 | HTA125 | Surface | Biolegend\* | 312802 |
| 143Nd | CD123 | 6H6 | Surface | Standard BioTools | 3143014B |
| 144Nd | FceR1 | AER-37 | Surface | Biolegend\* | 334602 |
| 145Nd | CD16 | 3G8 | Surface | Standard BioTools | 3145008B |
| 146Nd | CD8 | RPA-T8 | Surface | Standard BioTools | 3146001B |
| 147Sm | CD11c | Bu15 | Surface | Standard BioTools | 3147008B |
| 148Nd | CD34 | 581 | Surface | Standard BioTools | 3148001B |
| 149Sm | CD25 | 2A3 | Surface | Standard BioTools | 3149010B |
| 150Nd | pSTAT5 | 47 | Intracellular | Standard BioTools | 3150005A |
| 151Eu | CD14 | M5E2 | Surface | Standard BioTools | 3151009B |
| 152Sm | CD66b | 80H3 | Surface | Standard BioTools | 3152011B |
| 153Eu | pSTAT1 | 58D6 | Intracellular | Standard BioTools | 3153003A |
| 154Sm | CD10 | HI10a | Surface | Biolegend\* | 312202 |
| 155Gd | CD27 | L128 | Surface | Standard BioTools | 3155001B |
| 156Gd | p38 | D3F9 | Intracellular | Standard BioTools | 3156002A |
| 158Gd | pSTAT3 | 4/P-Stat3 | Intracellular | Standard BioTools | 3158005A |
| 159Tb | CD68 | Y1/48A | Intracellular | Standard BioTools | 3171011B |
| 160Gd | TLR8 | S16018A | Intracellular | Biolegend\* | 395502 |
| 161Dy | CD20 | H1 | Intracellular | Standard BioTools | 3161029D |
| 162Dy | Foxp3 | PCH101 | Intracellular | Standard BioTools | 3162011A |
| 163Dy | CD33 | p67.6 | Surface | Biolegend\* | 366602 |
| 164Dy | Iκβα | L35A5 | Intracellular | Standard BioTools | 3164004A |
| 165Ho | CD19 | HIB19 | Surface | Standard BioTools | 3165025B |
| 166Er | pNFkBp65 | K10895.12.50 | Intracellular | Standard BioTools | 3166006A |
| 167Er | CD38 | HIT2 | Surface | Standard BioTools | 3167001B |
| 168Er | CD73 | AD2 | Surface | Standard BioTools | 3168015B |
| 169Tm | CD45RA | HI100 | Surface | Standard BioTools | 3169008B |
| 170Er | CD117 | 104D2 | Surface | Biolegend\* | 313202 |
| 171Yb | pERK1/2 | D13.14.4E | Intracellular | Standard BioTools | 3171010A |
| 172Yb | CD15 | W6D3 | Intracellular | Standard BioTools | 3172021B |
| 173Yb | HLA-DR | L243 | Surface | Standard BioTools | 3173005B |
| 174Yb | CD4 | SK3 | Surface | Standard BioTools | 3174004B |
| 175Lu | pS6 | N7-548 | Intracellular | Standard BioTools | 3175009A |
| 176Yb | CD56 | HCD56 | Surface | Standard BioTools | 3176008B |
| 209Bi | CD11b | ICRF44 | Intracellular | Standard BioTools | 3209003B |
| \* Unlabeled purified antibodies were conjugated to metal isotopes using Maxpar X8 Antibody Labelling Kits (Standard BioTools) as per manufacturer's instructions. | | | | | |
Supplementary Table 3. Details of CyTOF antibodies used.
