# Supplementary material for: Inflammatory bone marrow signaling in pediatric acute myeloid leukemia distinguishes patients with poor outcomes
Source: Nat Commun. 2022 Nov 23;13:7186. doi: 10.1038/s41467-022-34965-4 (PMC9684530; doi:10.1038/s41467-022-34965-4)
Supplement: Supplementary file 3 — Reporting Summary [file 41467_2022_34965_MOESM3_ESM.pdf]

nature portfolio

Corresponding author(s): Hamid Bolouri, Soheil Meshinchi

Last updated by author(s): Oct 20, 2022



# Reporting Summary

Nature Portfolio wishes to improve the reproducibility of the work that we publish. This form provides structure for consistency and transparency in reporting. For further information on Nature Portfolio policies, see our Editorial Policies and the Editorial Policy Checklist.

## Statistics

For all statistical analyses, confirm that the following items are present in the figure legend, table legend, main text, or Methods section.

| n/a | Confirmed | |
|---|---|---|
| [ ] | [x] | The exact sample size (*n*) for each experimental group/condition, given as a discrete number and unit of measurement |
| [ ] | [x] | A statement on whether measurements were taken from distinct samples or whether the same sample was measured repeatedly |
| [ ] | [x] | The statistical test(s) used AND whether they are one- or two-sided<br>*Only common tests should be described solely by name; describe more complex techniques in the Methods section.* |
| [ ] | [x] | A description of all covariates tested |
| [ ] | [x] | A description of any assumptions or corrections, such as tests of normality and adjustment for multiple comparisons |
| [ ] | [x] | A full description of the statistical parameters including central tendency (e.g. means) or other basic estimates (e.g. regression coefficient) AND variation (e.g. standard deviation) or associated estimates of uncertainty (e.g. confidence intervals) |
| [ ] | [x] | For null hypothesis testing, the test statistic (e.g. *F*, *t*, *r*) with confidence intervals, effect sizes, degrees of freedom and *P* value noted<br>*Give P values as exact values whenever suitable.* |
| [x] | [ ] | For Bayesian analysis, information on the choice of priors and Markov chain Monte Carlo settings |
| [x] | [ ] | For hierarchical and complex designs, identification of the appropriate level for tests and full reporting of outcomes |
| [ ] | [x] | Estimates of effect sizes (e.g. Cohen's *d*, Pearson's *r*), indicating how they were calculated |

*Our web collection on statistics for biologists contains articles on many of the points above.*

## Software and code

Policy information about availability of computer code

| | |
|---|---|
| Data collection | RNA-seq FASTQ files were aligned to the GRCh38 human reference genome, using STAR v.2.4.2a and gene counts were generated using htseq-count (htseq version 0.6.1p1). QC and metrics analysis was performed using the Picard family of tools (vl.134). |
| Data analysis | The following free, open-source R (https://cran.r-project.org/) and Bioconductor (https://www.bioconductor.org/) packages were used for data analysis without modification or custom code:<br><br>ComplexHeatmap version 2.6.2<br>Cytofkit version 1.12.0<br>CytoML version 2.2.2<br>DESeq2 version 1.30.1<br>DMRcate version 2.4.1<br>edgeR version 3.32.1<br>enrichplot version 1.10.2<br>flowworkspace version 4.2.0<br>ggplot version 2 3.3.6<br>Gviz version 1.34.1<br>Hmisc version 4.4.2<br>IlluminaHumanMethylationEPICanno.ilm10b2.hg19 version 0.6.0<br>IlluminaHumanMethylationEPICmanifest version 0.3.0 |

Limma version 3.46.0
flowCore version 2.2.0
mclust version 5.4.7
minfi version 1.36.0
missMethyl version 1.24.0
pheatmap version 1.0.12
scater version 1.18.6
scMerge version 1.6.0
SingleCellExperiment version 1.12.0
Survival version 3.2.7
Survminer version 0.4.8

For manuscripts utilizing custom algorithms or software that are central to the research but not yet described in published literature, software must be made available to editors and reviewers. We strongly encourage code deposition in a community repository (e.g. GitHub). See the Nature Portfolio guidelines for submitting code & software for further information.

## Data

Policy information about availability of data

All manuscripts must include a data availability statement. This statement should provide the following information, where applicable:

- Accession codes, unique identifiers, or web links for publicly available datasets
- A description of any restrictions on data availability
- For clinical datasets or third party data, please ensure that the statement adheres to our policy

The raw RNA-seq data for the studies reported here have been deposited in the Database of Genotypes and Phenotypes (dbGaP, https://www.ncbi.nlm.nih.gov/gap/) under the study ID phs000465.v21.p8. Processed data are available at the National Cancer Institute's Genomic Data Commons (https://portal.gdc.cancer.gov/) under the TARGET-AML project. Selected clinical (e.g., age, EFS, OS, cytogenetic classification) and molecular features (e.g., KIT, RAS, NPM1, WT1, CEBPA, IDH1 mutations, and FLT3/ITD allelic ratios) were clinically available for patients included in the NCI/TARGET cohort and are included in the clinical data file available via the TARGET data matrix (ocg.cancer.gov/programs/target/data-matrix). CyTOF source data are provided with this paper.

## Human research participants

Policy information about studies involving human research participants and Sex and Gender in Research.

| | |
|---|---|
| Reporting on sex and gender | For these pediatric studies, only sex information was collected/reported. |
| Population characteristics | All patients are below the age of 29 years. Patients with certain conditions were excluded, as listed in the Recruitment Criteria below. |
| Recruitment | Eligibility criteria for COG Study AAML0531:<br><br>Ages Eligible for Study: up to 29 Years (Child, Adult)<br>Sexes Eligible for Study: All<br><br>DISEASE CHARACTERISTICS:<br><br>Newly diagnosed acute myeloid leukemia (AML)<br><br>Meets customary criteria for AML with ≥ 20% bone marrow blasts (by WHO classification)<br><br>Patients with < 20% bone marrow blasts and cytopenia or myelodysplastic syndromes (e.g., chronic myelomonocytic leukemia, refractory anemia (RA), RA with excess blasts, RA with ringed sideroblasts) are eligible provided 1 of the following criteria is met:<br><br>Karyotypic abnormality characteristic of de novo AML (t[8;21][q22;q22], inv[16][p13q22], t[16;16][p13;q22], or 11q23 abnormalities)<br>Unequivocal presence of megakaryoblasts (by WHO classification)<br>Isolated myeloid sarcoma (i.e., myeloblastoma or chloroma) allowed regardless of bone marrow results<br>Infants < 1 month of age with progressive disease* are eligible NOTE: *Infants < 1 month of age with AML may be given supportive care until it is clear that the leukemia is not regressing (i.e., the disappearance of peripheral blasts and the normalization of peripheral blood counts)<br>Patients with Down syndrome ≥ 4 years of age are eligible<br>No juvenile myelomonocytic leukemia<br>No Fanconi's anemia, Kostmann syndrome, Shwachman syndrome, or any other known bone marrow failure syndrome<br>No promyelocytic leukemia (M3)<br>No secondary or treatment-related AML<br>Matched family donor criteria (for patients with intermediate-risk or high-risk disease): |

HLA-A, -B, -C, and beta chain (-DRB1), identical or 1 antigen or allele mismatched by molecular high resolution technique
All available first-degree family members (parents and siblings) must be HLA typed
No syngeneic donors
Matched alternative donor criteria (for patients with high-risk disease):

HLA-A, -B, -C, and -DRB1, identical or 1 antigen or allele mismatched donor
HLA-A, -B, and -DRB1 4 of 6 antigen matched unrelated cord blood donor
Mismatched family member donor with ≥ 1 haplotype match or 5 of 6 antigen phenotypic match
PATIENT CHARACTERISTICS:

Not pregnant or nursing
Negative pregnancy test
Fertile patients must use effective contraception
PRIOR CONCURRENT THERAPY:

No prior chemotherapy, radiation therapy, or any antileukemic therapy

Topical or inhalation steroids for other conditions allowed
Intrathecal cytarabine given at diagnosis allowed
No other prior treatment for AML
No concurrent peripheral blood stem cell transplantation in patients with matched family donor

Eligibility criteria for COG Study AAML1031:

Ages Eligible for Study: up to 29 Years (Child, Adult)
Sexes Eligible for Study: All

Patients must be newly diagnosed with de novo acute myelogenous leukemia
Patients with previously untreated primary AML who meet the customary criteria for AML with >= 20% bone marrow blasts as set out in the 2008 World Health Organization (WHO) Myeloid Neoplasm Classification are eligible

Attempts to obtain bone marrow either by aspirate or biopsy must be made unless clinically prohibitive; in cases where it is clinically prohibitive, peripheral blood with an excess of 20% blasts and in which adequate flow cytometric and cytogenetics/ fluorescent in situ hybridization (FISH) testing is feasible can be substituted for the marrow exam at diagnosis
Patients with < 20% bone marrow blasts are eligible if they have:

A karyotypic abnormality characteristic of de novo AML (t(8;21)(q22;q22), inv(16)(p13q22) or t(16;16)(p13;q22) or 11q23 abnormalities
The unequivocal presence of megakaryoblasts, or
Biopsy proven isolated myeloid sarcoma (myeloblastoma; chloroma, including leukemia cutis)
Patients with any performance status are eligible for enrollment
Prior therapy with hydroxyurea, all-trans retinoic acid (ATRA), corticosteroids (any route), and IT cytarabine given at diagnosis is allowed; hydroxyurea and ATRA must be discontinued prior to initiation of protocol therapy; patients who have previously received any other chemotherapy, radiation therapy or any other antileukemic therapy are not eligible for this protocol
Exclusion Criteria:

Patients with any of the following constitutional conditions are not eligible:

Fanconi anemia
Shwachman syndrome
Any other known bone marrow failure syndrome
Patients with constitutional trisomy 21 or with constitutional mosaicism of trisomy 21 Note: enrollment may occur pending results of clinically indicated studies to exclude these conditions
Patients with any of the following oncologic diagnoses are not eligible:

Any concurrent malignancy
Juvenile myelomonocytic leukemia (JMML)
Philadelphia chromosome positive AML
Biphenotypic or bilineal acute leukemia
Acute promyelocytic leukemia
Acute myeloid leukemia arising from myelodysplasia
Therapy-related myeloid neoplasms Note: enrollment may occur pending results of clinically indicated studies to exclude these conditions
Pregnancy and breast feeding
Female patients who are pregnant are ineligible
Lactating females are not eligible unless they have agreed not to breastfeed their infants
Female patients of childbearing potential are not eligible unless a negative pregnancy test result has been obtained
Sexually active patients of reproductive potential are not eligible unless they have agreed to use an effective contraceptive

| | |
|---|---|
| | method for the duration of their study participation |
| Ethics oversight | Pediatric AML biological samples were collected with informed consent (and in accordance with the Declaration of Helsinki) from patients diagnosed with de novo AML and enrolled on Children's Oncology Group (COG) trials AAML0531 (NCT00372593)1, or AAML1031 (NCT01371981). Each protocol was approved by the National Cancer Institute's central institutional review board (IRB) and the local IRB at Fred Hutchinson Cancer Center (Protocol 9950). |

Note that full information on the approval of the study protocol must also be provided in the manuscript.

# Field-specific reporting

Please select the one below that is the best fit for your research. If you are not sure, read the appropriate sections before making your selection.

[x] Life sciences [ ] Behavioural & social sciences [ ] Ecological, evolutionary & environmental sciences

For a reference copy of the document with all sections, see nature.com/documents/nr-reporting-summary-flat.pdf

# Life sciences study design

All studies must disclose on these points even when the disclosure is negative.

| | |
|---|---|
| Sample size | For the Discovery RNA-seq data (n = 1275), as well as the validation RNA-seq data (n= 214) sample sizes were limited by the number of trial participants and the numbers of samples collected/assayed.<br><br>For the mechanistic studies, we used the R package 'ssizeRNA' and our Discovery cohort RNA-seq data, and estimated that a cohort size of n = 5 would allow us to detect > 2-fold gene expression changes in pathways consisting of > 10 genes with > 80% power. |
| Data exclusions | No data were excluded from the analysis. |
| Replication | Computational findings in the Discovery cohort ( n = 1275) were replicated and validated in an independent "Validation" cohort (n = 214). Subsequently, perturbation experiments were performed on 5 samples to verify the computationally inferred mechanisms. No other replication experiments were performed. |
| Randomization | Samples for the perturbation experiments were selected at random from a list of all qualifying samples (i.e. samples with the stated transcriptional and genomic characteristics). |
| Blinding | Blinding was not appropriate in the context of this study, because the only drug treatment assays performed were 'paired-sample' studies, comparing the same sample with and without treatment. |

# Reporting for specific materials, systems and methods

We require information from authors about some types of materials, experimental systems and methods used in many studies. Here, indicate whether each material, system or method listed is relevant to your study. If you are not sure if a list item applies to your research, read the appropriate section before selecting a response.

## Materials & experimental systems

| n/a | Involved in the study | |
|---|---|---|
| [ ] | [x] | Antibodies |
| [ ] | [x] | Eukaryotic cell lines |
| [x] | [ ] | Palaeontology and archaeology |
| [x] | [ ] | Animals and other organisms |
| [ ] | [x] | Clinical data |
| [x] | [ ] | Dual use research of concern |

## Methods

| n/a | Involved in the study | |
|---|---|---|
| [x] | [ ] | ChIP-seq |
| [ ] | [x] | Flow cytometry |
| [x] | [ ] | MRI-based neuroimaging |

## Antibodies

| | |
|---|---|
| Antibodies used | The table below provides the Label ,Target ,Clone, Cocktail ,Vendor, and Catalog Number of all antibodies used for CyTOF in comma separated format:<br><br>Label, Target, Clone, Cocktail, Vendor, Catalog Number<br>89Y, CD45, HI30, Surface, Standard BioTools, 3089003B<br>141Pr, CD3, UCHT1, Surface, Standard BioTools, 3141019B<br>142Nd, TLR4, HTA125, Surface, Biolegend*, 312802<br>143Nd, CD123, 6H6, Surface, Standard BioTools, 3143014B |

144Nd, FceR1, AER-37, Surface, Biolegend*, 334602
145Nd, CD16, 3G8, Surface, Standard BioTools, 3145008B
146Nd, CD8, RPA-T8, Surface, Standard BioTools, 3146001B
147Sm, CD11c, Bu15, Surface, Standard BioTools, 3147008B
148Nd, CD34, 581, Surface, Standard BioTools, 3148001B
149Sm, CD25, 2A3, Surface, Standard BioTools, 3149010B
150Nd, pSTAT5, 47, Intracellular, Standard BioTools, 3150005A
151Eu, CD14, M5E2, Surface, Standard BioTools, 3151009B
152Sm, CD66b, 80H3, Surface, Standard BioTools, 3152011B
153Eu, pSTAT1, 58D6, Intracellular, Standard BioTools, 3153003A
154Sm, CD10, HI10a, Surface, Biolegend*, 312202
155Gd, CD27, L128, Surface, Standard BioTools, 3155001B
156Gd, p38, D3F9, Intracellular, Standard BioTools, 3156002A
158Gd, pSTAT3, 4/P-Stat3, Intracellular, Standard BioTools, 3158005A
159Tb, CD68, Y1/48A, Intracellular, Standard BioTools, 3171011B
160Gd, TLR8, S16018A, Intracellular, Biolegend*, 395502
161Dy, CD20, H1, Intracellular, Standard BioTools, 3161029D
162Dy, Foxp3, PCH101, Intracellular, Standard BioTools, 3162011A
163Dy, CD33, p67.6, Surface, Biolegend*, 366602
164Dy, Iκβα, L35A5, Intracellular, Standard BioTools, 3164004A
165Ho, CD19, HIB19, Surface, Standard BioTools, 3165025B
166Er, pNFkBp65, K10895.12.50, Intracellular, Standard BioTools, 3166006A
167Er, CD38, HIT2, Surface, Standard BioTools, 3167001B
168Er, CD73, AD2, Surface, Standard BioTools, 3168015B
169Tm, CD45RA, HI100, Surface, Standard BioTools, 3169008B
170Er, CD117, 104D2, Surface, Biolegend*, 313202
171Yb, pERK1/2, D13.14.4E, Intracellular, Standard BioTools, 3171010A
172Yb, CD15, W6D3, Intracellular, Standard BioTools, 3172021B
173Yb, HLA-DR, L243, Surface, Standard BioTools, 3173005B
174Yb, CD4, SK3, Surface, Standard BioTools, 3174004B
175Lu, pS6, N7-548, Intracellular, Standard BioTools, 3175009A
176Yb, CD56, HCD56, Surface, Standard BioTools, 3176008B
209Bi, CD11b, ICRF44, Intracellular, Standard BioTools, 3209003B

* Unlabeled purified antibodies were conjugated to metal isotopes using Maxpar X8 Antibody Labelling Kits (Standard BioTools) as per manufacturer's instructions.

Validation

All antibodies against human immune markers used in this study have been previously validated by the manufacturer, as stated on their associated product web pages. Prior to their use as part of a CyTOF panel on study samples, all were tested at multiple titrations on bone marrow and whole blood samples with and without stimulation. Antibodies at optimal concentrations were combined into appropriate surface and intracellular cocktails and stored at -80°C to maintain consistency until use.

# Eukaryotic cell lines

Policy information about cell lines and Sex and Gender in Research

Cell line source(s)

HS-5 cells were a gift from the laboratory of Dr. Beverly Torok-Strob (Fred Hutchinson Cancer Center).

Authentication

None. HS-5 cells were obtained from the originating laboratory [BA Roecklein, B Torok-Storb, Functionally distinct human marrow stromal cell lines immortalized by transduction with the human papilloma virus E6/E7 genes, Blood, 85 (1995), pp. 997-1005]. The Torok-Strob laboratory performs regular assessments of the cells (e.g. PMID: 12184274).

Mycoplasma contamination

HS-5 cells were not tested for macoplasma contamination.

Commonly misidentified lines
(See ICLAC register)

NA

## Clinical data

Policy information about clinical studies

All manuscripts should comply with the ICMJEguidelines for publication of clinical research and a completedCONSORT checklist must be included with all submissions.

| | |
|---|---|
| Clinical trial registration | NCT01371981 (AAML1031) and NCT00372593 (AAML0531) |
| Study protocol | All data presented here are derived from the Children's Oncology Group {COG) AAML1031 and AAML0531 studies. All patient samples were obtained by member COG institutions after written consent from the parents/guardians of minors upon enrolling in the trial. |
| Data collection | AAML1031 opened on 6/20/2011. AAML1031 reached its accrual goal and closed to further patient entry on July 31, 2017. AAML0531 opened on September 7, 2006 and first reported it findings on January 13, 2015. |
| Outcomes | NA |

## Flow Cytometry

### Plots

Confirm that:

☒ The axis labels state the marker and fluorochrome used (e.g. CD4-FITC).

☒ The axis scales are clearly visible. Include numbers along axes only for bottom left plot of group (a 'group' is an analysis of identical markers).

☒ All plots are contour plots with outliers or pseudocolor plots.

☒ A numerical value for number of cells or percentage (with statistics) is provided.

### Methodology

| | |
|---|---|
| Sample preparation | AML cell cultures that were rested overnight and stimulated as in Methods were washed with cold PBS. Dead cells were labelled by incubation in Cell-ID™ Cisplatin (Standard BioTools) solution (5 µM in PBS) for 5 minutes at 4°C, then the reaction was quenched and cells washed with addition of an equal volume cold Media (RPMI + 25% FBS). Cells were then resuspended in Media and fixed by addition of and equal volume Fix I Buffer (BD Biosciences) and incubated for 15 minutes at 37°C. Cells were next barcoded using Standard BioTools Cell-ID 20-Plex Pd Barcoding Kit, as per manufacturer instructions. After washing, all samples from an individual were combined, and then resuspended in surface staining cocktail in Maxpar® Cell Staining Buffer (CSB, Standard BioTools) for 20 minutes at room temperature. Combined samples were washed then fixed with 1.6% paraformaldehyde in PBS for 10 minutes at room temperature. Samples were next incubated in ice-cold Perm Buffer III (BD Biosciences) for 1 hour at -20°C, then washed with CSB and then resuspended in intracellular staining cocktail in CSB for 20 minutes at room temperature. Samples were again washed and fixed with 1.6% paraformaldehyde in PBS for 10 minutes at room temperature, then stored in Maxpar® Fix and Perm Buffer (Standard BioTools) containing 125 nM Cell-ID™ Intercalator-Ir at 4°C until acquisition. On the day of acquisition, samples were washed with CSB and then cold ultrapure water, and kept at 4°C. Immediately before acquisition, samples were resuspended in cold ultrapure water containing 1/5th by volume EQ Four Element Calibration Beads (Standard BioTools) to at target concentration of 1 million cells/mL. |
| Instrument | Helios mass cytometer (CyTOF) (Standard BioTools) |
| Software | FCS files were normalized and randomized with CyTOF Software (version 7.0.8493) using Uniform Negative Distribution for the randomization and Median Bead Intensity with Passport EQ-P13H2302_ver2 without removing beads for the normalization.<br><br>Gating analysis was done using FlowJo version 10.7.1 (BD). |
| Cell population abundance | Not applicable. Cells were not sorted for this study. |

Gating strategy

Samples were first debarcoded by gating on cells triple-positive for Palladium isotopes (masses 104, 106, 108, and 110), and individual samples were exported as separate FCS files.

Within each stimulation condition sample, cells were gated to exclude beads (Ce140-), isolate singlets (using Ir191 and Event Length), and capture live cells (Pt198-). Immune cells were identified as live cells that expressed high levels of CD45 but lacked the stromal cell marker CD73. The strategy for identifying stem-cell-like AML blasts was to negatively gate out other immune subsets. Immune cells were divided into NK cells (CD56+CD3-) and T cells (CD3+CD56-). Within the CD3-CD56- portion, B cells were identified as those expressing CD19 and/or CD20. Non-B cells (CD19-CD20-) were gated to exclude Granulocytes (CD66b+CD15+), and within the non-granulocytes, Monocytes (expressing CD14 and/or CD16) were identified. CD14-CD16- (non-monocytes) were then divided by expression of CD34. Within CD34+ cells, a CD38+ CMP/GMP subset was identified (CMP/GMP AML). Within the CD34- cells, a CD123-CD33+ subset was identified (CD34- AML).

☒ Tick this box to confirm that a figure exemplifying the gating strategy is provided in the Supplementary Information.
