# Supplementary material for: Inflammatory bone marrow signaling in pediatric acute myeloid leukemia distinguishes patients with poor outcomes
Source: Nat Commun. 2022 Nov 23;13:7186. doi: 10.1038/s41467-022-34965-4 (PMC9684530; doi:10.1038/s41467-022-34965-4)
Supplement: Supplementary file 4 — Description of Additional Supplementary Files [file 41467_2022_34965_MOESM4_ESM.docx]

Descriptive legends for the Supplementary Data files

**Supplementary Data 1.** Genes differentially expressed between high-IL6/R pAML samples and healthy normal bone marrow.

**Supplementary Data 2**. Genes differentially expressed between high- and low-IL6/R pAML samples

**Supplementary Data 3.** 82 highIL6/R signature genes used to generate the heatmap in Figure 3B.

**Supplementary Data 4.** Targeted sequencing results for 181 high-IL6/R samples in Cluster 1 of Figure 3B, summarized in the Oncoprint of Figure 3C.

**Supplementary Data 5.** Frequencies of CD34+ and CD34- leukemic cell populations and the corresponding NBM CD34+ and CD34- populations summarized in Figure 4.

**Supplementary Data 6.**  Subtype-specific differentially expressed genes in genomic subtypes of high-IL6/R pAML samples.
